# Supplementary material for: The mental health and wellbeing of spouses, partners and children of emergency responders: A systematic review
Source: PLoS One. 2022 Jun 15;17(6):e0269659. doi: 10.1371/journal.pone.0269659 (PMC9200352; doi:10.1371/journal.pone.0269659)
Supplement: S1 Table — (DOCX) [file pone.0269659.s002.docx]

# **Supporting Information S1 Table. Full Table of Study Characteristics and Findings, Emergency Responder Families**

## **Quantitative Studies**

| **Author, Year, Country, (Reference no.)** | **Responder population (e.g., fire, police, ambulance)** | **Family Relation (e.g., spouse, child, parent)** | **Study Design** | **Sample size** | **Sub -Sample Sizes** | **Topic Area/ Summary** | **R.R** | **Mental health / Wellbeing Measures** | **Mental health/Wellbeing Summary Results** | **Statistical Findings** | **Additional Relevant Findings** | **Quality Assessment** |
| --- | --- | --- | --- | --- | --- | --- | --- | --- | --- | --- | --- | --- |
| **Anderson & Lo, 2011.**  United States  (54) | Police - law enforcement officers | Spouses | Self-report: survey | 1104 | Men = 86%  White = 64% | **Domestic Violence (DV)/ Interpersonal Violence (IPV)**  Hypothesises that police officers encountering stressful events on the job were likely to lose control at home and engage in IPV. The study also hypothesised that officers’ spillover authoritarian attitudes and negative emotions acquired in the workplace mediate stress’ effects on the occurrence of IPV. | 68% | **Gershon’s survey study of police stress and domestic violence in Baltimore, Maryland between 1997- 1999.** This included questions on psychological and physical stress and likely stressors, perceived current stress level, mechanisms used to cope with stress, and health conditions related to stress  **DV**: self-reported physical aggression towards partner.  **Task related stressful events**: 9-Item tool indicating how strongly they felt about: *making a violent arrest;* *shooting a person in the line of duty*; *being subjected to an internal investigation; responding to a chemical spill; responding to a bloody crime scene; learning one knows a victim personally; responding to a hostage situation; attending a police funeral; being exposed to blood or body fluids through a needle stick.*  **11- Item index of negative emotions** about whether the participant was ever; tired at work despite adequate sleep; moody, irritable, or impatient over small job-related problems; withdrawn as a result of unrelenting job demands on time and energy; given to feelings of futility, negativity, or depression about work; inefficient at work; physically, emotionally, or spiritually depleted; less able to resist illness because of work; less interested in pursuing fun activities because of work; unable to care about problems and needs of members of the public; unable to concentrate at work; present at work only because its required. | - Women and non-white worse than white men in self-reported physical aggression. - For white male officers, authoritarian aggression and negative emotions accounted for the physical aggression – whilst for female African American officers, authoritarian spillover did not have a significant contribution. Physical violence was more related to negative feelings about their work. | - 9% admitted to losing control and becoming physically aggressive. Of those: 28% were African American men; 27% were African American women; 41% were white men; 4% were white women. - Significant positive correlations found between 3 independent variables: experiencing stressful events, authoritarian spillover, negative emotions, and DV. - **Correlations:** show that female and non-white respondents likelier to engage in physical aggression against intimate partners. - The highest correlation was found between years of employment and current rank (r=.56). - **Logistic regression**: at all stages of analysis, gender and race were significant predictors: men 60% less likely than women to carry out assault; white 59% less likely than non–white. - Significant increase in likelihood of IPV accompanying an increase in authoritarian spillover and negative emotions – each increase linked to a 9% increase in likelihood of each variable. Increase in negative emotions linked to 13% increase in IPV likelihood. | The maladaptive strategy known as authoritarian spillover serves police in a way to cope with task related stress. Here, the mediating roles of authoritarian spillover and negative emotions indicate that among police officers, increased likelihood of IPV reflects the diminished capacity to handle task related stress.  Because the task related stress variable had only a small effect on physical aggression, it was evident that authoritarian spillover and negative emotions worked as mediators and significant factors in domestic violence in police officers’ homes. | 4 |
| **Brimhall et al., 2016**  United States  (41) | Police officers | Romantic partners/ spouses | Cross -sectional: self-report questionnaire | 302 | 85 were in a committed relationship and provided information of their partner  58 partners agreed to participate (of these, 54 were male police officers with female partners; 4 were female officers with their respective partners) – since there weren’t enough female officers, the analysis was limited to the 54 male officers and their partners.  Mean age = 39, SD = 7.06  Partners mean age = 38.06, SD = 7.32 | **Couple relationships:**  Association between attachment, communication, and satisfaction. | 41% overall,  68% RR of the couples | **Brief Accessibility, Responsiveness, and Engagement Scale:** (BARE; Sandberg, Busby, Johnson, & Yoshida, 2012): 12-item scale that measures how accessible, responsive, and engaged the participants view themselves and their partner to be. Items are scored on a 5-point Likert scale ranging from 1 (never true) to 5 (always true).  Cut-off scores for individuals’ self-report of their own attachment behaviours are (a) mild distress: 25; (b) moderate distress: 22; and (c) severe distress: 19. Cut-off scores for individuals’ view of their partner’s behaviours are (a) mild distress: 25; (b) moderate distress: 20; and (c) severe distress: 17.  **Revised Dyadic Adjustment Scale:** (RDAS); Busby, Christensen, Crane, & Larson, 1995) assesses relationship satisfaction (14-Item). Couples who score below a 48 are considered distressed. Internal reliability scores were adequate for both officers (a =0.93) and their partners (a =0.89).  **Communication Patterns Questionnaire:** (CPQ; Christensen & Sullaway, 1984): 35 items + 9-point likert scales assessing the individuals perception of how people handle conflict both during and after an argument. | - Results suggest that secure behaviours increase constructive communication, decrease demand-withdrawal, and increase satisfaction. - It appears that male behaviours have a stronger influence on the findings than female partners. Clinicians are encouraged to consider interventions that not only increase secure bonds but engage the male partner. - **RDAS** scores for officers ranged from 13 to 67 with 29% considered distressed. - Partners ranged from 25 to 66 with 24% classified as distressed. There were no significant differences based on rank or division. - **Summary**   (a) A secure attachment bond, one that nourishes and strengthens both partners’ physical and emotional well-being, has consistently been shown to act as a reliable source of support to help minimize the effects of stress; However, building a secure attachment bond requires engaging in meaningful interactions where each partner is accessible, responsive, and engaged, often this is impeded by the police culture of keeping things to themselves to protect their partners.  (b) Male’s behaviour is a stronger predictor of positive communication; partners’ perceptions of officers’ attachment were positively associated with how likely they were to engage in constructive communication. | **Is attachment linked with constructive communication, demand/withdrawal, and marital satisfaction?**   - Significant differences did not emerge between officers and their partners on any variable. - Greater mutual constructive communication as reported by officers was significantly positively linked with both officers’ reports of their own attachment (b =0 .39, p < .01) and with their perceptions of their partners’ attachment (b = 0.35, p <0.05) - Mutual constructive communication as reported by partners was not significantly associated with officers’ perceptions of their own and of partners’ attachment. - Only a partner effect was found in that the partners report of mutual constructive communication was significantly positively associated with partners perceptions of officers’ attachment (b = 0.67, p<.0.1). This suggests that when partners perceive that officers are more securely attached, partners are more likely to report mutual constructive communication. - The model accounted for 49.2% of the variance in officers’ reports of mutual constructive communication and 57.3% of the variance in partners’ reports. - Officers’ self-reported demand/withdrawal communication was significantly negatively linked with officers’ perceptions of both their own (b =0 .41, p < .01) and their partners’ attachment (b = 0 .35, p < .05) - Partners’ self-reported demand/withdrawal communication was significantly positively linked with officers’ perceptions of their own attachment (b = .47, p < .01). - Conversely, partners’ self-reported demand/ withdrawal communication was significantly negatively linked with officers’ perceptions of their partners’ attachment (b = 0 .43, p < .01) - Partners’ self-reported demand/ withdrawal communication was significantly negatively associated with partners’ reports of officers’ attachment (b =0 .59, p < .01), such that when partners perceived officers to be more securely attached, partners reported fewer demand/withdrawal behaviours. - This model accounted for 46.1% of the variability in officer-reported demand/withdrawal communication behaviours and 43.7% of the variability in partners’ reports of this construct. - Greater marital satisfaction was linked with both officers’ reports of their own (b = 0.33, p<0.01) and their partners’ attachment (b= 0.39, p<0.01) – when officers reported that both they and their partners were securely attached they were more likely to report greater marital satisfaction. - Partners reports of marital satisfaction were significantly positively linked to both partners reports of their own (b = 0.38, p<0.01) and of officers’ attachment (b = 0.47, p<0.01). When partners reported that both were securely attached, they were also more likely to report higher marital satisfaction. - The model accounted for 55.4% of variance for officer reported marital satisfaction and 75.1% for partners. | “Rather than focusing on interventions to reduce stress, clinicians should consider therapeutic methods to help police (and to some extent their partners) realise that engaging emotionally with their partners, even around difficult conversations, will do more to “protect” them emotionally than keeping things to themselves”. | 5 |
| **Comer et al, 2014**  United States  (60) | Police | Children | Cross-sectional self-report questionnaires | 83 | Average age of child was 11.2, SD = 3.6. 81.9% of children were white, Caucasian. | **Mental health of children whose parents or relatives participated in the Boston manhunt.** | 41.6% | **UCLA PTSD Reaction Index (RI):** Parent-Report Symptom Scale was used to assess child PTSD symptoms.  **Strengths and Difficulties Questionnaire (SDQ):** (1) emotional symptoms, (2) conduct problems, (3) hyperactivity/inattention, (4) peer problems, and (5) prosocial behavior. A total difficulties score is generated by adding subscales 1–4.  The survey also collected data on children’s presence at the finish line during the blast, as well as details of children’s specific exposures to the subsequent manhunt. | - Having a relative in the manhunt retained significant predictive associations with PTSD symptoms, emotional symptoms, and hyperactivity/inattention, whereas it no longer showed a significant predictive association with total difficulties. Child age did not moderate any of the significant effects of having a relative in the manhunt. - Children with close relatives in law enforcement or the Armed Forces who participated in the manhunt carried a particularly heavy mental health burden. - 1/9 children sampled with family in the manhunt had “likely PTSD”. Even after accounting for children’s initial marathon blast exposure and children’s own manhunt exposure - having a relative participating in the manhunt was significantly linked with elevated PTSD symptoms, emotional symptoms, and hyperactivity/inattention. - Higher proportions of youth with relatives in the manhunt saw uniformed persons not typically found in civilian neighbourhoods, saw officers with guns drawn related to the manhunt, heard and saw manhunt-related gunshots/explosions, saw manhunt-related blood, had an officer knock on their door and enter/search their home, and knew the slain officer or injured transit officer. - Roughly half of youth with relatives in the manhunt saw a heavier police presence in their neighbourhood, as well as uniformed persons not typically found in civilian neighbourhoods, whereas less than one-third of youth without relatives in the manhunt had such experiences. - The proportion of youth hearing or seeing manhunt-related gunshots/explosions, seeing manhunt related blood, and having an officer knock on their door and enter/search their home was 3–5 times higher for youth with relatives in the manhunt. - Clinical outcomes, on average, fell within normative ranges, although there was considerable variability, and children with relatives in the manhunt reported significantly greater posttraumatic stress, emotional symptoms, hyperactivity/inattention, and total difficulties than children without a relative in the manhunt. - The proportion of youth with likely PTSD was 5.7 times higher among youth with relatives in the manhunt than youth without. | - Manhunt Exposures Tally was higher among youth with a relative in the manhunt (M = 2.61, SD = 2.7) than among youth without (M = 1.45, SD = 1.7), F(1,441) = 24.05, P < .001. - Among youth with relatives in the manhunt, the largest manhunt exposure effects on PTSD symptoms were for youth who knew the slain officer (d = 3.43), saw blood related to the manhunt (d = 2.49), or knew the injured transit officer (d = 1.96). - Children fear during the manhunt that a loved one might be seriously hurt was higher among youth with a relative in the manhunt (M = 5.31, SD = .35) than youth without (M = 3.84, SD = .17), F(1,441) = 14.28, P < .001. - There was a mediation effect for PTSD symptoms (a × b = 1.984, SE = 0.826, z = 2.401, P < .05), emotional symptoms (a × b = 0.217, SE = 0.102, z = 2.139, P < .05), and total child difficulties (a × b = 0.490, SE = 0.237, z = 2.067, P < .05), indicating that children with a relative in the manhunt expressed more fear that a loved one might be seriously hurt, and in turn displayed more PTSD symptoms, emotional problems, and total difficulties. - Living in the zone of greatest manhunt activity had a significant main effect on fear of a loved one being hurt (P < .05 across models) and on PTSD symptoms (P < .01). However, it did not moderate effects of having a relative in the manhunt on outcomes (e path, P > .10 for all models) or effects of having a relative in the manhunt or fear of a loved one being hurt (d path, P > .40 for all models). Because the d path was not significant, living in the zone of greatest manhunt activity was also not able to moderate the mediated effect of having a relative in the manhunt on clinical outcomes via fear of a loved one being hurt (d∗b, P > .40 for all models). |  | 5 |
| **Craun et al., 2015.**  United States  (46) | Police – Internet Crimes Against Children personnel (ICAC) | Friends and family affected by ICAC work | Self-report: survey | 600 | Gender:  Male = 66%, female = 24.5%, did not report = 9.5%  Race: African American = 1.7%, Asian = 0.7%, Latino-Hispanic = 4.1%, White/Caucasian = 80.1%, did not report race = 13.3%  Marital Status: Married = 69.4%, not married = 20.4%, did not report marital status = 10.2%  Children = 71.6, no children = 19.8, did not report = 8.6%  Average years in law enforcement = 15.4, SD = 7.9; Average years in child exploitation = 5.2, SD = 4.4.  *more details on education, role type, military experience available. | **ICAC work impact on family and friends** | n/a | To measure how participants viewed their work on the taskforce as impacting their outside personal lives, they asked the open-ended question: **How has this work affected your relationships with family, children, and friends?** The first and third authors read all the open-ended answers, developed 11 categories, and independently coded each question. The kappa score measuring inter-rater agreement was calculated and at 0.80.  **Bride’s Secondary Traumatic Stress Scale (STS) (Bride et al. 2003).** The Secondary Traumatic Stress Scale measures the frequency of STS symptoms in the last seven days. Cronbach’s alpha was 0.94.  ***Control Variables:*** They explored utilisation of different coping styles using subscales from the COPE scale (Carver et al. 1989). Coping styles examined were: 5 point scales measuring coping through denial, social support, and positive reinterpretation.  4-point scales to measure social support from supervisors and co-workers.  **Marlowe-Crowne** **Social Desirability Scale**, Short Version.  Also controlled for were measures of:   - how often respondents view child pornography - how difficult it was for respondents to view different types of child pornography - If they drank alcohol and if their use of alcohol had increased in the past year - Tobacco use - Gender | How Do Professionals Serving on ICAC Task Forces View Their Work as Impacting Their Relationships with Their Family and Friends?   - The most frequent response was that participants felt there was no change in their relationships (27.2 %). - Other common answers were that respondents were more distrusting of others (24.3 %) and more withdrawn from their relationships (14.2 %). - One out of 10 respondents provided answers that indicated they felt their work with the ICAC taskforce improved their relationships.   ***Conclusions***   - Some of the findings were similar to what has been previously reported by other researchers, such as the work causing officers to withdraw from family and being distrustful of others. - Respondents who stated that their job made them more distrustful of others or indicated they could not talk to friends and family about their time in the ICAC did not have higher STS scores; more compelling predictors of symptoms of STS included irritability, social withdrawal, and marital difficulties. - ICAC taskforce members who described their job as positively affecting their relationships with family had lower STS scores than those who did not mention any positive aspects. - An interaction term between gender and STS score was found to be significant in predicting comfort in expressing intimacy with one’s own children. STS appeared to increase the discomfort in expressing intimacy with one’s own children more in males than in females. - A positive finding that implies better outcomes for ICAC personnel is that STS was not related to comfort expressing intimacy with one’s spouse or partner. Additionally, neither the frequency nor self-reported difficulty interacting with disturbing media was related to difficulty expressing intimacy with a spouse or children. - It was noted that male ICAC members expressed higher comfort expressing intimacy with spouses as compared to female taskforce members. | - STS scores were examined among those who mentioned their job effects their relationships compared to those who did not. Those who said ICAC work had no impact on their relationships (t (476) = 8.26, p < .001) or that it made their relationships better (t (476) = 3.46, p < .001) had lower STS scores. - Those who reported irritability (t (476) = −2.72, p < .001), marital difficulties (t (476) = −4.32, p < .001), and withdrawing from friends and family (t (476) = −6.48, p < .001) had significantly higher levels of STS. - Reporting that the job made respondents more distrustful of others’ intentions (t (476) = −1.46, p = .14) or that respondents felt they could not speak with friends and family about their job (t (476) = −0.07, p = .95) had no impact on STS scores.   *Can STS Scores Predict Comfort Expressing Intimacy with Spouse and Children in an ICAC Taskforce Sample?*   - The answer was split. When examining comfort expressing intimacy with children, the average score was 2.33 (SD = 1.11), suggesting that respondents generally did not believe they were having difficulty in this area. - An initial model illustrated that the average STS score was related to closeness with one’s own children. When an interaction term for gender and mean STS score was entered into the model, a significant relationship (p < .05) was found. Namely, men with higher STS scores had steeper slopes in their discomfort in expressing intimacy with their own children as compared to female taskforce members’ interactions with their own children; women’s level of discomfort in expressing intimacy with children also increased as their STS increased, just not at the same pace as the men in this sample. - Endorsing denial as a coping mechanism was related to more discomfort in expressing intimacy with one’s children (p < .05). - Average frequency and average self- reported difficulty with child pornography were not related to difficulty expressing intimacy with one’s children (p = .12 and p = .47, respectively). - Overall, respondents reported that they felt comfortable expressing intimacy with their spouses and partners (M = 4.14, SD = 0.98). When investigating a possible relationship between STS scores and comfort level with intimacy with one’s spouse or partner, while controlling for other variables related to STS, the relationship between STS and comfort with spousal intimacy was not statistically significant (p = .07). - Also, as with the results regarding intimacy with children, the average frequency where one interacts with disturbing media, such as child pornography, along with the self-reported difficulty in witnessing child pornography, were not related to intimacy difficulties with one’s spouse or partner (p = .37 and p = .56). |  | 3 |
| **Davidson et al., 2006.**  Australia  (35) | Police officers | Partners | Self -report questionnaires | 103 couples: police officers and their spousal partners | Police officers: 102 = male, 1 = female  Spouses: 102 = female; 1 = male.  Police officers were aged between 36 and 63 years (M = 50.6, SD = 5.3), and their spouses were 2 years younger (M = 48.6, SD = 6.0).  These couples had lived together for an average of 25 years.  The highest ranked participant was at commander level; widely distributed in both rural and metropolitan areas. | **Mental health of police officers and partners:**  Partners were hypothesised to demonstrate patterns of psychological adjustment like the officers. | 24% | ***Trauma Exposure:*** Frequency of exposure to each of 17 critical incident types. Following the critical incident exposure list, officers were asked: ‘Which event during your service has been the most upsetting? (Please describe)’.  A second list of traumatic events was presented to both officers and partners. This list was intended to capture the non-duty-related trauma exposure of officers and the trauma history of partners. The events on the list included being seriously physically attacked or raped, involvement in a natural disaster, experiencing a life-threatening accident, serious abuse or neglect as a child, or suffering great shock as a result of one of these events happening to someone close to the respondent.  ***Posttraumatic Stress Symptomatology: The PCL -*** The Posttraumatic Stress Disorder Checklist (PCL; Weathers, Litz, Herman, Huska, & Keane, 1993) Items are rated on a 5-point Likert scale and total scores can range from 17 (no symptoms) to 85 (severe symptoms).  ***Psychological Wellbeing: The GHQ-28-*** The General Health Questionnaire (GHQ-28) (Goldberg & Williams, 1988). Items are rated on a 4-point Likert scale, yielding total scores from 0 (no symptoms) to 84 (severe symptoms), and scores from 0 to 21 for severity of symptoms within each of the 4 domains. | - Main Findings: The presence of PTSD in trauma survivors fosters psychological disturbance in their intimate partners. This outcome was sustained after the prior trauma exposure of partners was controlled. The PTSD cluster of avoidance and emotional numbing symptoms in officers played the most significant role in this relationship. PTSD avoidance-numbing was associated with the level of severity of all the psychological variables in partners. - Inverse relationship between police and partner hyperarousal symptoms. Possibly, when the officer appears aroused, such as irritable, excessively jumpy, or demonstrating poor concentration, the partner may consciously reduce their own arousal to preclude any amplification of these symptoms in the officer - Based on PCL scores, nearly one third of the officers and almost 14% of the partners demonstrated PTSD. - Regression analyses identified that, after controlling for partners’ prior trauma exposure, characteristics of officer psychological adjustment — particularly avoidance-numbing symptoms — were significant predictors of poorer psychological adjustment in their partners      - *Trauma Exposure:* Officers utilised a range of responses to the list of duty-related traumatic events; Workplace stress featured prominently in this other category, and included problems with managers/supervisors, inadequate support from command, structural changes within the police service, and problems with subordinate staff. - Both partners and officers reported extensive trauma exposure in their private lives. Almost 72% of officers and 61% of partners had experienced between one and seven of the different types of traumatic events contained in the list of non-work traumas. Officers had also experienced more events than their partners — 24% endorsing four or more incidents on the list — compared with about 9% of partners.   ***Conclusions***   - A sizeable congruence can be observed between the scores of officers and partners. - Psychological disturbance predictors: officer avoidance/ numbing was positively associated with partners’ scores on all 4 GHQ subscales. - There was a negative association between officers’ hyperarousal and both partners’ somatic symptoms and anxiety/sleep disturbance. - Partner depression was predicted by officers’ somatic symptoms, social dysfunction, depression, and avoidance/numbing symptoms. - Traumatic stress predictors: officer anxiety/sleep disturbance and avoidance-numbing symptoms both predicted traumatic stress in partners, whereas lower levels of officer hyperarousal were associated with partner PTSD. - Officer anxiety/sleep disturbance and avoidance-numbing symptoms both predicted partner re-experiencing symptoms and there was a negative association between officer hyperarousal and partner reexperiencing. - Partner avoidance-numbing was positively associated with officer somatic symptoms and officer avoidance-numbing, but it was negatively associated with officer hyperarousal. - Finally, partner hyperarousal was predicted by officer avoidance-numbing, and lower levels of officer hyperarousal predicted increased hyperarousal symptoms in partners. | Psychological Outcomes and significant correlations   - GHQ total: officers: M=21.9, SD = 14.1; Partners: M = 21.3, SD = 13.1, r=.38, p<.01 - GHQ somatic concerns: officers: M=6.0 SD =4.8; Partners: M = 6.2, SD = 4.8, r=.29, p<.01. - GHQ anxiety/sleep disturbances: officers: M=6.5 SD =5.1; Partners: M = 6.1, SD = 5.4, r=.27, p<.01. - GHQ social dysfunction: officers: M=7.6 SD =3.0; Partners: M = 7.4, SD = 2.9, r=.32, p<.01. - GHQ severe depression: officers: M=1.9, SD =3.1; Partners: M = 1.7, SD = 3.1, r=.42, p<.01. - PCL total: officers: M=35.5, SD =17.1; Partners: M = 32.0, SD = 14.0, r=.34, p<.01. - PCL re-experiencing: *non-significant* - PCL avoidance/numbing: officers: M=2.0, SD =1.0; Partners: M = 1.7, SD = 0.8, r=.50, p<.01. - PCL hyperarousal: *non-significant* - Using a score of 44 as a cut-off for screening for posttraumatic stress disorder, 32% of the 97 officers who completed the PCL, and 15 (13.5%) of the 89 partners who completed the PCL produced scores that exceed the criterion.   Predictors of Partners’ Psychological Adjustment:   - Officer avoidance/numbing sig predicted symptom severity: R^2^=.39, t=4.08, p<0.001; somatic concerns: R^2^=.33, t=4.36, p<0.001; anxiety/insomnia: R^2^=.35, t=3.29, p<0.01; social dysfunction: R^2^=.19, t=2.23, p<0.05; severe depression: R^2^=.38, t=2.36, p<0.05. - Officers hyperarousal negatively associated with symptom severity: R^2^=.39, t= -2.76, p<0.01; somatic concerns: R^2^=.33, t= -3.27, p<0.01; anxiety/insomnia: R^2^=.35, t= -2.69, p<0.01. - Partners’ social dysfunction was predicted only by officers’ avoidance/numbing which accounted for 19% of the variance, however, because the presence of a multivariate outlier, this model was no longer significant at α = .01. - Partner depression was predicted by officers’ somatic symptoms t=2.18, p<0.05; social dysfunction: t=2.08, p<0.05; depression: t=2.30, p<0.05; avoidance/ numbing: t=2.36, p<0.05; together these predictors accounted for 38% of the variance.   Predictors of Partners traumatic stress   - Traumatic stress in partners was predicted by: officer anxiety/sleep disturbance: t=3.65, p<0.001; and avoidance-numbing symptoms: t=3.26, p<0.01; whereas lower levels of officer hyperarousal were associated with partner PTSD:, t= -3.04, p<0.01; This model accounted for 45% of the variance in Partner PTSD. - Partner re-experiencing symptoms predicted by: officer anxiety/sleep disturbance: t= 3.34, p<0.001; and avoidance-numbing symptoms: t= 2.77, p<0.01; negative association between officer hyperarousal and partner reexperiencing t= -2.28, p<0.05; Together, these predictors accounted for 55% of the variance. - Partner avoidance-numbing was positively associated with officer anxiety/ insomnia: t= 2.96, p<0.01; and officer avoidance-numbing: t= 3.50, p<0.001; but it was negatively associated with officer hyperarousal: t= -2.44, p<0.05; Together, these predictors accounted for 38% of the variance. - Partner hyperarousal was predicted by officer avoidance-numbing: t= 2.04 p<0.05; and lower levels of officer hyperarousal predicted increased hyperarousal symptoms in partners: t= -3.05, p<0.01. These 2 predictors jointly accounted for 53% of the variance. | An implication of this study is that partners of traumatised, symptomatic police officers may well need support themselves, and whether this need extends to the children in these families warrants investigation. | 6 |
| **Duarte et al., 2006.**  United States  (61) | Police, and firefighters, and EMTs. | Children of first responders (FR) (following 9/11) | Self-report questionnaire | 8236 children/ adolescents | Ages 9-21  53.1% female; most reported ethnicity = Latino = 40.1%, African American = 27.9%, white = 13.4%, Asian = 12.8%, mixed/other=5.7%.  In the total sample 15.5% had at least one responder family member. | **Mental health:**  Posttraumatic stress disorder (PTSD) in children whose parents worked as first responders during 9/11 (EMTs or firefighters) | 69% in 4-5^th^ graders and 95.8% in 6-8^th^ graders | **PTSD assessed by the PTSD screening module of the Diagnostic Interview Schedule for Children (DISC) DISC Predictive Scales (DPS)** (Lucas et al.,2001).  **Information about family members’ occupations** was obtained. Multiple responses were acceptable, and they included police officers (PO), firefighters (FF) and emergency medical technicians (EMT).  **Children’s exposure to trauma was measured** by (a) previous exposure, defined as exposure to traumatic situations before September 11 (Saltzman et al., 1999); (b) attendance in a Ground Zero Area school; (c) direct exposure, defined as two or more of the following types of exposure: personally witnessing the attack, being hurt in the attack, being in or near the cloud of dust and smoke, having to be evacuated to safety, or being extremely worried about the safety of a loved one; (d) family exposure, defined as having a family member killed or injured in the attack, or witnessing the attack but having escaped unharmed; and (e) high TV exposure, child spent a lot of time watching attack coverage on the TV (Hoven et al., 2005). | - High rate of probable PTSD among children with EMT family members in a representative sample of NYC public school students 6 months after the WTC attack – this is explained by a combination of their exposure to the WTC attack and socio-demographic characteristics. - The highest rate of probable PTSD (18.9%) occurred in children with EMT family members. The rate among children who had police family members (10.6%) was similar to that among children without any FR (10.1%), whereas children with firefighter family members had the lowest prevalence of probable PTSD (5.6%). Groups were roughly comparable regarding attendance in Ground Zero schools, direct, and TV exposure. - However, those with EMT family members or at least two of the FR professions in their families had high levels of prior exposure. Family exposure was less frequent among children with only police or no FRs in their family. Most children with EMT family members were non-White (92.4%); about 2/3 of the FF children were White (62.5%). | Logistic regression analysis of probable PTSD   - When being the child of a FR was considered, children from all groups were less likely to have probable PTSD compared to children who had an EMT family member (reference group), although the results were only statistically significant for children of firefighters and marginally significant for those without a first responder in their family (p=.0516). - When controlling for different types of exposure children with FFs in their family were still less likely than children with EMTs in their family to have probable PTSD. However, the introduction of demographic variables resulted in the adjusted odds ratio for this comparison being only marginally significant (p=.07). | More detailed analysis revealed that the possible differences in risk of probable PTSD among EMT children, compared to children in families with no FR was mostly explained by differences in WTC attack exposure.  Compared to children with FFs in their families, a great part of the elevated risk among children with EMTs in their families could be attributed to demographics.  The “heroism” associated with being a FF might also be important in helping FFs’ children cope with stress. it might help to develop interventions to foster resilience based on children’s positive appraisal of a family member’s occupation. | 4 |
| **Erwin et al., 2005**  United states  (59) | Police Officers (who were married or live with a domestic partner and have had reports filed against them from 1992 - 1998) | Spouses | Police records | 106 cases |  | **Domestic Violence (DV) / Intimate Partner Violence (IPV)** | n/a | Data were abstracted from all DV/IPV incidence reports filed with the department’s Internal Investigation Division (IID) from 1992, when a standardized report form was introduced, to June 1998. | - Officers accused of IPV were more likely to be members of a minority, on the force more than 7 years and assigned to a high crime district. No major differences on intake baseline personality (MMPI) scores were noted between cases and controls. - Most of the final dispositions of the cases resulted in cases closed due to unsupported testimony from victims | - Most reports cited actual assaults (n = 81, 77%). - The types of assault included both physical assault (battering) as well as sexual assault. Fewer incidences involved harassment (n = 6, 9%), such as refusal to leave the premises, and stalking (n = 3, 3%), defined as telephone misuse, following the victim to work, and following the victim’s car. - 12 of the cases involved threats, including threats of bodily harm with a weapon (most often a gun, and in one instance a knife). - 23% of the accused officers had a history of at least one prior IPV report on file with the police department and 5% had two or more priors. - The majority of accused officers (n = 68, 64%) were immediately suspended from duty, 28 (26%) had a protection order issued against them, and 18 (17%) were immediately arrested. However, the final administrative disposition of the majority (92%) of cases resulted in no action, usually because of a lack of testimony or unsupported evidence (61%), lack of physical evidence (31%), and conflicting testimony (1%). | Reports were most often made against male officers (n = 89) with most of these filed by the officer’s wife (n = 43, 48%), followed by the officer’s ex-wife or former girlfriends (n = 24, 27%), and by their present girlfriend (n = 22, 25%). When female officers were the accused, most reports were either filed by their present boyfriends (n = 6, 35%), present girlfriends (n = 6, 35%), ex-husbands and former boyfriends (n = 3, 18%), and by their husbands (n = 2, 12%). | 5 |
| **Gibson et al., 2001**  United States  (53) | Police | Spouse/ partner | Cross-sectional survey | 596 | 434 (72.8%) white, 162 (27.2%) minorities.  94 (15.8%) had high school diplomas, 334 (56.0%) had some college, 146  (24.5%) had college degrees, and 22 (3.7%) had graduate degrees.  33 (5.5%) were officer trainees, 325 (54.5%) were officers, 26 (4.4%)  were agents, 74 (12.4%) were detectives, 93 (15.6%) were sergeants, and 44  (7.4%) were lieutenants or higher in rank. | **Domestic Violence (DV)** | 44.2% | **Police stress questionnaire was developed:**  Strain measure consisting of job dissatisfaction and negative work-related events, measures for both anger and depression to represent negative effects, and measures of social support and spiritual coping that Agnew (1992) and others (Piquero and Sealock, 2000) suggest may condition the relationship between strain, negative effects, and violence. | - Logistic and OLS regression analyses indicate that occupational strain has an indirect relationship with DV through negative affective measures of anger and depression. Strain/negative affect-alleviating factors, i.e., social support and spiritual coping, did not influence DV. | - Of the 596 officers in the sample, 44 (7.4%) reported getting physical with their spouse or significant other, 48 (8.1%) reported getting physical with their child(ren), and 69 (11.6%) reported getting physical with their pet(s). In all, 106 officers (17.8%) report engaging in at least one of the three domestic violence behaviours. - Strain exerted a positive and significant effect (a, = .42) on DV. Officers experiencing more strain were significantly more likely to report engaging in domestic violence. When in the equation by itself, strain accounted for 3% of the proportional reduction in chi-square. - Once anger and depression were entered into the model, the effect of strain became nullified, and the effects of anger (8,= .52) and depression (a, = .49) both exerted positive and significant effects on domestic violence. Angrier officers and more depressed officers were significantly more likely to report engaging in domestic violence.   Logistic Regression:   - Anger had the strongest effect (DR = .61) on domestic violence. Officers that were angrier were more likely to report engaging in domestic violence. Also, depression had a positive and significant effect (DR = .44). Officers who felt more depressed were more likely to report engaging in domestic violence. - In congruence with the risk factor literature on domestic violence, physical abuse had a positive and significant effect (DR = .45) on domestic violence, indicating that officers reporting being physically abused by their parent(s) in childhood were more likely to report engaging in domestic violence | Strain was positively and significantly associated with migraine headaches (r = .19, p < .05), chronic lower back pain (r = .29, p < .05), heart disease (r = .14, p < .05), chronic insomnia (r = .25, p < .05), and high blood pressure (r = .09, p < .05) | 5 |
| **Haddock et al., 2016.**  United States  (40) | Firefighters | Partners/ marriage/ relationships | Cross-sectional survey | 1,456 | 49 (3.4%) were women, consistent with national rates of women in the fire service.  Most personnel described race/ethnicity as White, non-Hispanic (74.2%).  Among the women, 72.4% held the position of firefighter, 18.3% were in the company officer position, and 10.2% “other.”  Among the men in the sample, 68.4% firefighters 22.2% company officers, 5% chief officer positions; 4.4% “other”. | **Marriage/ divorce:**  Looks at relationship status, history of divorce, key health outcomes/ demographic variables affecting the above | 97% for FIRE study and 94.4% for F2F study | 2 epidemiologic cohort studies within the U.S. fire service conducted by the Center for Fire, Rescue and EMS Health Research at the National Development and Research Institutes, Inc., and the University of Texas School of Public Health.  **Firefighter Injury and Risk Evaluation Study (FIRE Study; EMW2007-FP-02571)** designed to prospectively assess injury and cardiovascular risk factors.  **The Fuel to Fight Study (F2F Study; EMW-2009-FP-01971)** was conducted nationally to examine the impact of wellness programs in fire departments, assessment of nutrient intake in career firefighters.  ***Body composition:*** Fat and muscle ratio etc. using a portable stadiometer etc.  ***Physical activity/fitness:*** The Self Report of Physical Activity (SRPA) questionnaire (Jackson & Ross, 1997).  ***Tobacco :*** Behavioural Risk Factor Surveillance System (Centers for Disease Control Prevention, 2009) and the Department of Defense’s Survey of Health Related Behaviors Among Active Duty Military Personnel (Bray et al., 2009).  ***Alcohol use:*** OH surveys  ***Anxiety and depression:*** Current depressive symptoms were assessed with the **Center for Epidemiological Studies Short Depression Scale** (CES-D 10; Irwin, Artin, & Oxman, 1999). Those endorsing 4 or more items in the negative direction are in the range of concern for depression.  ***Marital status***: Options: of married, divorced, widowed, separated, never married, a member of an unmarried couple. History of divorce was assessed with response options of never, once, twice, three or more times. | - Prevalence of divorce among male firefighters was similar to the general population. In contrast, age-standardized prevalence of current divorce among female fighters (32.1%) was more than three times that for females in the general population (10.4%). - Prevalence of ever having divorced was substantially higher among female (40.0%) compared with male firefighters (24.4%). - Married firefighters were less likely to report heavy alcohol use or depression. - These findings suggest that the rate of divorce among male firefighters is similar to that found in the general public. In contrast, female firefighters report high rates of divorce.   ***Age/Gender Standardized Prevalence***   - For men, 77.0% were married compared with the U.S. population prevalence of 57.5%. The age-standardized prevalence of currently divorced male firefighters was 11.8% compared with 9.4% of the U.S. population. Among women, the adjusted prevalence of current marriage was 42.6% for the fire service compared with 55.4% in the U.S. population sample. - The age standardized prevalence of divorce among women in the fire service was 32.1% compared with 10.4% in the U.S. population.   ***Prevalence of Ever having Divorced***   - Among married firefighters, prevalence of ever having divorced was higher among females compared with males (27.3% vs. 19.6%). When all firefighters were examined, prevalence of ever having divorced was substantially higher among female firefighters (40.0% vs. 24.4%). - Overall, 25% of firefighters reported being ever divorced. | ***Current Marital Status***   - 45% of men and 64.3% of women in the youngest age group (19-29 years) reported having never been married. - Overall odds of being currently married were significantly lower in women compared with men (Odds Ratio [OR] = 0.25; 95% Confidence Interval [CI] = [0.14, 0.45]; p < .0001). - In contrast, the odds of currently being divorced was much greater in women compared with men across age groups (OR = 3.97; 95% CI = [2.08, 7.56]; p < .0001). Gender was not significantly related to the odds of being part of an unmarried couple.   ***Multivariate Model Predicting Current Marital/Partnered Status***  Age (F = 46.18; p < .001) and gender (F = 12.33; p < .001) were significant predictors in the final multivariate model, with older and male firefighters more likely to be married/partnered. Among the behavioural health factors, waist circumference (F = 0.19; p < .003), heavy drinking (F = 12.45; p < .001), and a previous depression diagnosis (F = 14.95; p < .001) remained as significant predictors in the multivariate model. |  | 4 |
| **Halbesleben et al., 2010**  United States  (67) | Police | Married co-workers - spouses of police officers who are also police | Cross-sectional | n=621 Police couples  n=369 other working adult couples | The police officer sample was primarily male (n = 942), with an average age of 42.28 (SD = 10.44) years.  The sample was primarily White (n = 695.  From the larger sample of 1,103, only officers who were married or had a live-in partner and whose spouse/partner held a job were included in the analysis, yielding a final sample size of 621. | **Explores how married co-workers cope with work-related stress when compared to a typical dual-career employees.**  Married co-workers may utilise different coping strategies because of the additional resources provided by their spouse etc. | 68% | Spouse workplace and occupation were assessed by asking four sequential questions. Only participants who were married or had a live-in partner were included in the analysis.  **Social support** was measured using a one-item measure (“I feel that I can rely on support from my spouse”). This item was scored on a five-point, Likert-type scale from strongly agree (1) to strongly disagree (5); lower scores indicate higher perceived support.  **Coping strategies** were assessed with a 10-item scale adapted from previous work of Beehr, Johnson, and Nieva (1995) and Billings and Moos (1981). All of the items were assessed on a four-point frequency scale from never (1) to always (4).  The number of children living at home with the officer was included as a control variable. Tenure and gender also controlled for. | - There is a positive relationship between spousal support and active coping, which is strengthened when the couple share an occupation or workplace. - For avoidant coping, those that share an occupation or workplace with their spouse show a negative relationship between spousal support and avoidant coping; again that is stronger in those who do share an occupation than for those who do not share an occupation or workplace with their spouse. | - After adding the control variables, the main effect of both spousal support (beta = .22, p < .01) and married co-worker status (beta = .08, p < .05) were significantly associated with active coping; the addition of those variables also led to a significant increase in the variance accounted for in active coping (deltaR2 = .11, p < .01). - When the interaction between spousal support and married co-worker status was added to the regression, it was significant (beta = .38, p < .01) and its addition led to a significant increase in the variance accounted for in active coping (delta R2 = .03, p < .05). - Avoidant coping: in sample 1 after adding the control variables the main effect of both spousal support (beta =-.27, p<.01) and married co-worker status (beta =-.15, p<.05) were significantly associated with avoidant coping - the addition of these variables also led to a significant increase in the variance accounted for in avoidant coping (dedltaR2 = .11, p < .01). - When the interaction between spousal support and married co-worker status was added to the regression, it was not significant (beta = -.25, ns) and its addition did not lead to a significant increase in the variance accounted for in avoidant coping (deltaR2 = .01, ns). | These findings were consistent with the idea that those people with greater support resources are in a better position to engage in active coping; those who are in a married co-worker relationship may have resources that are particularly relevant and thus may be in an even better position to engage in active coping.  With regard to avoidant coping, the predicted negative relationship between social support and avoidant coping in both samples. This confirmed the prediction that those with fewer resources are more likely to engage in avoidant coping because they do not have the resources to invest in active coping. | 6 |
| **Hoven et al., 2009**  United States  (62) | First Responders (FR): fire fighters, police officers, and Emergency Medical Technicians(EMT)/ World Trade Center (WTC) evacuees | Children, ages 9-16 living in the household with a FR | Cross country and cross-sectional questionnaires | 900 families (total of 2700 individual family member)  Control group= 180 families, not WTC evacueematched with WTC evacuee =180 | Not reported | **Mental health of children of FR:**  In a representative sample of New York City (NYC) public school children assessed 6 months after the WTC attack | Not reported | **Mental health**  Child  **Diagnostic Interview Schedule for Children**, Version IV—DISC-IV (Shaffer et al. 2000)  **DISC Predictive Scales** (DPS) (Lucas et al. 2001)  **Child tobacco use** adapted from questions from the National Survey for Parents and Youth from NIDA.  Adult:  PTSD, depression, alcohol, substance use: **Composite International Diagnostic Interview—CIDI** (Wittchen 1994) PTSD checklist (PCL) (Weathers et al. 1993) Beck Depression Inventory II (BDI) (Beck et al. 1996) K-10 (Kessler et al. 2002).  **Exposure to potentially traumatic events:** Work related:  **CIHQ** (Marmar et al., 1996)  Mass violence: **WTC-BOE- Survey Questionnaire- child version** (Hoven et al., 2002b)  Individual:**Stressful life events** (Gray et al., 2004)  **Other risk/protective factors being measured:**  Child: Demographics, Intelligence, Personality, family environment, Parenting, Social Support, Appraisal of Parental Exposure, Disaster Preparedness, Prior trauma, Family and Media Exposure, Health effects, School performance, Service need and utilization, Coping strategies, Loss and bereavement, Discrimination, stigma, and prejudice, Perspectives on the future.  Adult: Demographics, Personality, Family Environment, Prior trauma, Family and Media Exposure, Health effects, Service need and utilization, coping strategies, Loss and bereavement, Discrimination, stigma, and prejudice, Perspectives on the future a Includes violent and nonviolent events. | - The study shows seemingly elevated rates of psychopathology among children of WTC evacuees. - Children of NYC First Responders (police officers, EMTs, and fire fighters) displayed a complex pattern of response to the WTC attack with differential PTSD prevalence between branches of service with children of EMTs displaying highest PTSD, police and then firefighters. - The results show the impact of having a family member or a parent who died or was an evacuee on children’s psychopathology, especially probable PTSD and major depression, separately for children who had direct exposure to the WTC attack and those not exposed to the WTC attack. | **Prevalence:**  Probable PTSD and depression, among NYC public school students in grades 4–12, whose family members were (or were not) in the WTC at the time of the attacks (N = 8,236):   - The results indicate that a child’s direct exposure to the attack, and a family member’s exposure to the WTC attack, each independently contribute to an increased risk for developing probable PTSD or major depression. - Among those children who were not exposed to the WTC attack, the lowest rate of probable PTSD was found among those who did not have family members who were in the WTC during the attack (6.8%); the highest rate of probable PTSD was found among children whose family members had died during the WTC attack (17.6%). Relatively higher rates of PTSD were found among those with direct exposure to the WTC attack, given the same type of family member exposure status. - The highest rate of PTSD was found among those children who was direct exposed to the WTC attack and had a family member die during the attack (36.4%). A weaker but similar relationship was found for probable major depression.   Probable PTSD and depression, post-WTC attack among NYC Public School students in grades 4–12, with and without FR in the home (Children who had EMTs only, police officers only and fire fighters only living at home are included. Data are not shown for children who had two or more First Responders living at home (N = 237)):   - Children of EMTs as family members had the highest rate of probable PTSD (15.1%), followed by those with police officers as family members (8.1%), who were very similar to children not having any family member being First Responders (7.5%), while children with fire fighters as family members who had the lowest prevalence of probable PTSD (2.9%). - The distribution for children with direct exposure to the WTC attack has a similar pattern**.** For probable major depression, those with family member EMTs also had the highest rates compared to other groups. But the rates of probable major depression for those in families with fire fighters were not lower than those families with police officers and those families without FR**.** - This all suggests a different impact on child mental health as related to specific First Responder jobs held by the parent. | Authors’ comment: For children of FR, the experience of a mass violent traumatic event to which a parent had been exposed, may affect children simply by their knowing that their parents will be on the front lines of a future mass violent event such as terrorism. | 5 |
| **Johnson et al., 2005.**  United States  (58) | Police Officers (who were married or live with a domestic partner) | Spouses/ partners | Cross-sectional survey | 413 | Males n=351  Females n=62 | **Domestic violence(DV)/ violence exposure:**  Examines relationship between violence exposure and domestic violence among police officers with the expectation that the relationships would be both direct and mediated. The mediation factors included burnout, authoritarian spillover, alcohol use, and department withdrawal. The most powerful of these was burnout and authoritarian spillover. | Not reported. | Survey of 333 items covering a variety of work-family issues (for detailed information on how the sample was obtained, see Beehr et al., 1995)  **Burnout:** Burnout that captures detachment and hardening of emotions— “*external burnout*.” Respondents were asked how often during the last 6 months prior to the survey did they *(a) treat civilians like objects, (b) feel callous towards citizens, (c) worry that the job was hardening them, (d) feel unconcerned about the welfare of civilians, (e) feel that working with people all day is a strain, and (f) feel that working directly with people is too much stress.* The response format for these items consisted of a 7-point Likert type scale (Cronbach’s alpha = .85).  **Authoritarian spillover:** Items: “*I like to do things by the book at home,” “My job conditioned me to expect to have the final say on how things are done in my household,” “I can’t shake the feeling of being a police officer when at home,” “I have become overly critical at home due to the police job,” “I hold my family’s behaviour to a high standard because I am a police officer,” and “I catch myself treating my family the way* *I treat the civilians.”.* 7-point Likert type scale anchored at the end points with “strongly disagree” and “strongly agree” (Cronbach’s alpha = .74)  **Alcohol Use:** 7-point Likert scale, with 3 items: *“Did you have periods when you could not remember what happened when you were drinking?” “Did you ever worry or feel guilty about your alcohol consumption?” and “Did you ever drink more than you planned?”* (Cronbach’s alpha = .85).  **Violence exposure:** To determine the officers’ exposure to violence, they were asked how often in the last 6 months they experienced 7 specific violent events: (Cronbach’s alpha = .81).  **Spouse Violence:** Respondents reported on their out of control behaviour against co-workers, clients, and family members. The present study focuses on their response to 1 question, that is, *how often in the last 6 months had they gotten out of control and behaved violently towards their spouse.* The response format for this question consisted of a 7-point Likert scale. | - In the path model used to explore relations among predictors and outcome variables they treated *exposure to violence* in the course of policing as a potential direct and indirect cause of *spousal violence.* The mediating variables in the model are *psychological and behavioral: external burnout, authoritarian spillover, departmental withdrawal, and alcohol use.*  This model ended up accounting for 17% of the variance in spousal violence. - The effect of violence exposure on domestic violence is a mediated process. First, violence exposure matters most if it leads to spousal violence through external burnout and authoritarian spillover. - Pervasive impact of burnout on all mediators and the outcome factor (domestic violence). Authoritarian spillover was also an important factor. - Although a third of the officers say that alcohol use is a problem in their lives, it does not play a powerful role in spousal violence. The effects of burnout and authoritarian spillover far outweigh that of alcohol use.   Authors note surprise at the lack of direct association between violence exposure and spousal violence. | **Statistical analysis**   - 4 separate mediational chains: The first chain involved the indirect effect of exposure to violence on spouse violence with external burnout as the sole mediator. This indirect effect was significant, b_1_b_2_ = .03, z = 2.08, p< .05. - The second mediational chain, exposure to violence on spouse violence with alcohol as the sole mediator was marginally sig: b_1_b_2_ = .01, z = 1.70 p< .09. - The third mediational chain comprised the effect of exposure to violence on external burnout, the effect of external burnout on authoritarian spillover, and finally the effect of authoritarian spillover on spouse violence. This indirect effect was significant: b_1_b_2_b_3_ = .02, z = 3.35 p< .001. - The fourth mediational chain included the effect of exposure to violence on external burnout, the effect of external burnout on alcohol use, and finally the effect of alcohol use on spouse violence. This indirect effect was only marginally significant, b_1_b_2_b_3_ = .01, z = 1.94, p<.06. - As the direct effect of departmental withdrawal on spouse violence was not significant, they did not evaluate mediational chains involving this effect. |  | 4 |
| **Karaffa et al., 2015.**  USA  (30) | Police officers | Spouses | Cross-sectional | 82 officers and 89 spouses | 95% of the officers were male (n =78), and almost 98% (n =88) of the spouses were female.  The average age of the officers completing the survey was 39 (SD = 8.67), and the average age of spouses completing the survey was 36 (SD =8.49).  The sample was predominately Caucasian (89% of the officers and 87% of spouses).  The modal number of years married to a police officer, as reported by spouses, was 1–5 years (32.6%). Officers and spouses had both been married an average of 1 time, with a minimum of 1 and a maximum of 4 times. | **Impact of police work on spouses:**  Determining the types of difficulties evident in police marriages.  Spouses reported feeling pride about being married to an officer, they also noted financial concerns, WFC, and law enforcement-specific stressors, such as negative public attitudes toward police. Officers and spouses reported relying on friends and family for support more than on professional sources. | Unable to calculate | 2 separate but similar needs of assessment surveys to identify the types of difficulties that police officers and spouses must endure.  Potential sources of support were also examined.  The needs assessments included demographic questions as well as items relating to familial stressors, perceived conflict, and utilization of various sources of support.  **Survey:**  85 items in 4 categories: demographics (10), perceived level of conflict (9), stressors and perceived support (45), and resources used (21). We asked spouses to respond to 88 similar questions, also in these categories, with 3 additional items about stressors and perceived support. These items were worded similarly but focused on spouses’ experiences. | - Spouses tended to indicate the greatest perceived conflict related to financial concerns and emotional intimacy. Regarding financial concerns, 36% of spouses indicated a high level of conflict over finances and almost 35% indicated a medium level of conflict. - Approximately 24% of spouses reported high levels of conflict regarding emotional intimacy and almost 26% reported a medium level of conflict. - Relatively fewer spouses reported conflict over physical intimacy or use of alcohol. Approximately 16% of spouses reported high levels of conflict over physical intimacy, and only slightly more than 3% of spouses reported high levels of conflict over their partners’ use of alcohol. | **Perceived level of conflict:**   - Spouses (M = 1.67, SD = 0.81) reported significantly higher conflict regarding the kind of attention their spouses gives them, when compared to officers (M =1.45, SD = 0.63), t (165) = 2.02, p =.045, d = 0.31.   **Stressors and Sources of Support:**  **Financial concerns:**   - Most spouses either agreed or strongly agreed with the statement ‘‘I have to work in order to meet our family’s financial needs.’’ Over half reported being troubled by the fact that officers had to work extra jobs to meet the family’s financial needs. When officers and spouses were asked if both partners had to work in order to meet financial needs, the mean score obtained by spouses (M = 3.79, SD = 1.24) was significantly higher than the score obtained by officers (M = 3.39, SD = 1.22), t(169) = 2.10, p = .037, d = 0.33, indicating that spouses were more likely to admit that they had to work.   **Work-Family-Conflict (WFC)**   - Spouses reported the most concern about officers missing important family events (53.9%) and giving most of their energy to the job (49.5%). - 26% of spouses indicated that they felt as if they had to handle family problems on their own. The mean score obtained by spouses (M =- 3.11, SD = 1.18) on the item ‘‘it is difficult for me when my spouse has to choose between his/her job and our family’’ was significantly higher than officers’ responses to the item ‘‘choosing between my job and my family is difficult for me’’ (M = 2.41, SD = 1.15), t(167) = 3.94, p < .001, with a large effect size (d = 1.02).   **Personality**   - 33% of spouses reported that their significant other is likely to yell at a family member if he or she experienced a stressful event at work. - 31% agreed that their spouse expects to have the final say in matters, and approximately 30% agreed that their spouse releases work stress on the family. - 16% reported that their spouse was controlling and overbearing. Spouses were more likely to report that their significant other yells at family members (M = 2.54, SD = 1.37) than officers were to admit yelling at family members (M = 2.17, SD = 1.00), t(169) = 2.00, p =.047, d = 0.31.   **Public attitudes**   - Over 68% of spouses agreed that the public has higher demands for police officers and their families than others, although officers (M =- 4.18, SD = 0.69) perceived significantly greater public demands than spouses did (M = 3.78, SD = 0.93), t(169)= 3.25, p = .001, d = 0.49. - Over 30% of spouses reported that negative attitudes and rude treatment from the public creates stress for them or their family.   **Communication/ emotions:**   - Most spouses agreed that they can discuss and resolve family problems with their significant other, and their significant other can talk with family members about work stress (85.4% and 84.3%, respectively). - Likewise, over 77% indicated that they can share intimate thoughts and feelings. Relatively fewer (16.8%) respondents admitted that work stress has affected their spouse’s interest or pleasure in physical intimacy. Despite these positive findings, more than 1/3 of spouses agreed that they feel as if their spouse has part of his or her life closed off to them. - Some spouses indicated concern over communication and officers’ peer relationships. Over 38% perceived that their spouse talks more openly with colleagues. Spouses (M = 2.97, SD =1.26) were significantly more likely than officers (M = 2.49, SD = 0.99) to agree that they talked more openly to colleagues, t (167) = 2.69, p = .008, d = 0.42.   **Departmental support**   - Spouses perceived mixed support from the department. Although 54% agreed that the department supports their family, only approximately 11% agreed that the department provides adequate sources of stress relief to officers and fewer than 7% agreed that the department provided ways for spouses or family members to deal with stress. - Spouses (M = 2.58, SD = 0.93) were more likely to agree that the department provided adequate stress relief outlets for their employees than officers (M = 2.28, SD = 0.92), t (168) = 2.11, p = .037, d = 0.32.   **Family pride in the profession:**   - 86.6% of spouses reported that they were proud of their significant others’ career in law enforcement. The mean score obtained by spouses (M = 4.73, SD = 0.52) was significantly higher than the score obtained by officers (M = 4.21, SD =0.77), t(169) = 5.13, p < .001, d=0.79, suggesting that officers reported less perceived pride from their spouses. - Furthermore, almost 72% of spouses agreed that their children were proud of their parents’ career in law enforcement. - Almost 80% of spouses agreed that they felt optimistic about their significant other’s future as a police officer, and approximately 66% said that they would still choose to marry a police officer, even knowing how it affects the family. - However, spouses’ responses to whether they would still choose to marry a police officer (M = 3.86, SD = 1.18) were significantly lower than scores of officers who were asked if they would still choose to be a police officer (M = 4.20, SD=0.95), t(168) = 2.01, p = .046, d = 0.32.   **Spirituality**   - Most respondents in the spouse group (89.2%) reported that their spiritual beliefs gave them strength. Spouses (M = 4.51, SD= 0.71) were significantly more likely to report that their spiritual beliefs gave them strength than officers (M =4.07, SD = 0.95), t (168) = 3.39, p = .001, d = 0.52. | **Resources Used**  Spouses reported that their family was most likely to receive support from family members (89.9%) and friends (82%). ~50% of spouses indicated that their family received support from other officers and almost 44% received support from other police families.  Over 46% of spouses indicated that their family received support from clergy. Only 7.9% (n =7) of the spouses reported that their family received support from professional counsellors, and only 1 respondent reported that her family utilized support groups.  Chi-square analyses indicated that there were no significant differences between officers and spouses in their endorsement of the family support items. Spouses were most likely to report receiving support from family members (77.5%) and friends (75.3%) to deal with the stress of being a police spouse. 27% reported receiving support from other police spouses, and approximately 11% receive support from other officers. Most spouses reported that their spiritual beliefs give them strength and over 46% reported that their family receives support from clergy, only about 21% report that they receive support individually. Furthermore, only 4.5% (n = 4) of spouses reported receiving support from professional counsellors and only 1 spouse reported receiving support from support groups to deal with the stressors of being a police spouse. | **3** |
| **Kishon et al., 2020**  Israel  (64) | Fire, paramedics, non-traditional first responders (FRs) | Spouse and child | Cross-sectional case control | 108 families | families of active-duty male FRs: fire- fighters (n=72), paramedics/Emergency Medicine Technicians (EMT)(n=33), and non-traditional FRs (n= 3)—and control families (n=100) | **Parental occupational exposure impact on child mental health** | N/A | Parent Psychopathy  **PCL-C** (Weathers et al. 1993) – sum of scores of 17 items, 5-point likert scale  **Beck Depression Inventory** **for Primary Care** (BDI-PC) (Beck et al. 1996) – total symptom severity score (0-21)  **10-item Kessler Psychological Distress Scale** (K10) (Kessler et al. 2002).  Child psychopathy  Symptoms of PTSD related to any traumatic event reported in the **DISC DSM-IV PTSD criterion A scale**.  Generalized anxiety disorder (GAD), separation anxiety disorder (SAD), agoraphobia (AGO), panic disorder (PD), major depressive disorder (MDD), oppositional defiant disorder (ODD), and conduct disorder (CD) were assessed with the **DISC Predictive Scales (DPS)**  Both parent and child asked to report on child’s symptoms  Latent class analysis (LCA) examined the comorbidity among the 8 DPS disorders – 3 classes – no disturbance, internalising symptoms, externalising symptoms  Functional impairment | - Maternal exposure was associated with a greater number of symptoms in the child of generalized anxiety, panic disorder, depression, and oppositional defiant disorder, and with increased odds of comorbid internalizing symptomatology - In the final models the association between paternal First Responder (FR) status and PTSD in child became non-significant; however, the relationship between maternal cumulative exposure to work-related events and SAD, AGO, and likelihood of belonging to the externalizing class was only marginally significant. - Parental psychopathology was significantly associated with their children’s outcomes. Maternal and paternal PTSD were associated with children’s PTSD; paternal PTSD was also associated with GAD, SAD, AGO, MDD, and ODD. - Maternal and paternal K10 scores were associated with MDD. Maternal K10 scores were associated also with GAD, AGO and externalizing outcomes, such as ODD, CD, and likelihood of belonging to the externalizing class); paternal K10 scores were also associated with other internalizing outcomes, namely PTSD, SAD, PD, and likelihood of belonging to the internalizing class. - Maternal and paternal BDI scores had the opposite effects on PTSD and CD; paternal BDI scores were negatively associated with PTSD, and positively associated with CD, while maternal BDI scores showed the reverse relationship. - Negative family satisfaction and negative parent-child relationship were associated with SAD, ODD, and likelihood of belonging to internalizing and externalizing classes. In addition, negative family satisfaction was associated with PTSD and CD, while negative parent-child relationship was associated with GAD, AGO, and MDD. - No significant relationships with the outcomes were observed for negative monitoring/attachment. | Paternal FR status associated with Separation anxiety disorder (Estimate -1.35 SE=0.15) | Post hoc analysis showed that having a father working as a FR was mainly associated with a higher chance of reporting recurrent excessive distress when anticipating or experiencing separation from parents, reluctance/refusal to go to sleep without being near the parents, and physical symptoms (e.g., headaches, stomach aches, nausea) when separation occurs or is anticipated  Non-significant relationship of paternal FR status and child PTSD - authors argue may be due to predominance of firefighters in the sample who in previous studies children have shown to have lower PTSD levels if firefighter parent. | 4 |
| **King & DeLongs, 2014.**  Canada  (42) | Paramedics | Cohabitating spouse/ partners | Longitudinal: survey design | 87 paramedics; 87 partners | Paramedics:  All worked full time hours and similar shift patterns.  Average years on the job = 15.2 (SD 7.7).  82=Caucasian, 4 = Asian, 1 = Hispanic.  Mean age = 42.1 year (SD 8.3; range 27 – 62 years).  Male (n=71).  Partners:  79= Caucasian, 5= Asian, 2=First Nations, 1= Latin American.  Mean age = 41.3 years (SD 9.2, range 24 – 74 years),  Female (n=75).  72 spouses = employed outside the home (56 full-time).  66 couples were married, with 55 couples having at least 1 child living in the home.  Average length of relationship = 13.1 year (SD 8.7), average length of cohabitation =11.6 years (SD 8.6). | **Relationships**  Examines two different coping responses, rumination and interpersonal withdrawal as they relate to occupational stress and interact in the home setting. | 15.6% | Information was collected from paramedics and spouses for a period of 4 consecutive work shifts (according to the paramedic’s schedule).  Paramedics answered questions 3x daily: (T1) within 1/2 hours of waking (in reference to “the day so far”);  (T2) immediately after work (in reference to their time spent “at work”); and  (T3) before bed (in reference to the period of time “since last entry”).  Spouses answered questions twice daily at T1 and T3 only.  4-item version of the **Perceived Stress Scale (**PSS-4; Cohen, Kamarck, & Mermelstein, 1983) was completed by paramedics at T2. In the current sample of paramedics, internal reliability estimates were moderate across days, with a mean of .67.  **Occupational burnout:** Maslach Burnout Inventory – Human Services Survey 3 main dimensions: emotional exhaustion, depersonalization, and reduced personal accomplishment (Maslach et al., 1997). Daily measure derived from (MBI-HSS; Maslach, Jackson, & Leiter, 1996) and included at T2. Average alpha =.78.  **Coping.:** At T3: rumination and interpersonal withdrawal.  State rumination was measured using 3 items from the **Rumination-Reflection Questionnaire** (RRQ; Trapnell & Campbell, 1999).  Interpersonal withdrawal was measured using the **interpersonal withdrawal subscale from the Brief Ways of Coping Inventory** (Lee-Baggley et al., 2005).  Daily measures of rumination and withdrawal demonstrated good internal reliability over the 4 days, with average alphas of .81 and .67, respectively.  **Marital tension**: Assessed 2x a day: at T1 for daily baseline measures and at T3 as a marker of daily dyadic functioning. Paramedics and their spouses were asked, “How much tension or conflict has there been with your spouse/romantic partner?” Participants responded on a 5-point Likert scale. | - Significant associations were observed between paramedics’ work stress and subsequent rumination and withdrawal on the part of paramedics. While paramedics’ work stress was not associated with spouses’ rumination, paramedics’ burnout at work was associated with increases in spouses’ interpersonal withdrawal. - Regarding the role of these coping responses in daily marital functioning, paramedics’ rumination and spouses’ withdrawal were associated with increased marital tension over the 4-day period. On days when spouses withdrew more from the relationship, the associations between paramedics’ rumination and both partners’ reports of marital tension were greater.   **Hypothesis 1***: higher levels of work stress and burnout as reported by paramedics would predict higher levels of paramedic rumination.*   - Paramedics’ T2 perceived stress at work demonstrated a significant and positive association with T3 rumination suggesting that paramedic rumination increased on days when work stress was greater. This effect occurred across the day, such that higher rumination was reported at home subsequent to earlier reports of work stress. - T2 burnout did not impact T3 rumination for paramedics, nor did paramedics’ T2 work measures have any impact on spouses’ T3 rumination)   **Hypothesis 2:** *paramedics’ work stress and burnout would predict higher levels of interpersonal withdrawal for both paramedics and spouses.*   - **P**aramedics’ T2 perceived stress at work was associated with increased T3 withdrawal, whereas T2 burnout had no such effect. - T2 burnout as reported by paramedics demonstrated a significant and positive association with spouses’ T3 withdrawal, whereas the effect of paramedics’ T2 perceived stress was nonsignificant for spouses. Significant effects occurred across the day, such that higher work stress and burnout reported by paramedics were associated with subsequent reports of paramedics’ rumination and spouses’ withdrawal (respectively).   **Hypothesis 3:** *on days when paramedics ruminated more, the impact on marital tension for both partners would be exacerbated if spouses withdrew more from the relationship.*   - Paramedics’ T3 rumination displayed significant and positive associations with both paramedics’ and spouses’ concurrent T3 marital tension, controlling for earlier reports of T1 marital tension. - T3 marital tension was associated with spouses’ concurrent T3 interpersonal withdrawal for spouses, but not for paramedics. The interaction between paramedics’ rumination and spouses’ withdrawal was significant at p < .01 for both paramedics and spouses. - Paramedics’ burnout interacted significantly with spouses’ withdrawal to predict increases in both partners’ T3 marital tension. | **Descriptive measures and t tests:**   - Bivariate correlations were calculated using average scores across days. Paramedic T2 stress and T2 burnout were significantly correlated (r =.50, p < .001). - Significant correlations were also observed between T1 and T3 marital tension for both paramedics (r =.66, p < .001) and spouses (r =.29, p < .05), supporting the inclusion of T1 reports of marital tension as controls in models predicting T3 marital tension. - Paramedic and spouse T1 reports of marital tension were significantly correlated (r =.53, p < .001), as were paramedic and spouse T3 reports of marital tension (r =.49, p < .001). - Significant bivariate relationships were observed between T3 rumination and T3 interpersonal withdrawal for both paramedics (r =.47, p < .001) and spouses (r =.53, p < .001). - A significant correlation was not observed between paramedic rumination and spouse withdrawal, or between paramedic withdrawal and spouse rumination. - Significant bivariate association between T3 paramedic rumination (as IV) and T3 spouse withdrawal (as DV; beta= .13, SE =.06, p < .05); and between T3 spouse withdrawal (as IV) and T3 paramedic rumination (as DV; beta = .17, SE =.08, p < .05). 53% of the variance in T2 burnout occurred between couples, compared with 65% of the variance in T2 stress, 13% of the variance in T3 rumination, 16% of the variance in T3 withdrawal, and 22% of the variance in T3 marital tension. - Lagged effects were also examined among key variables, but none were significant. Previous day’s T2 stress and burnout did not significantly predict next day’s marital tension (either T1 or T3), nor did previous day’s T3 rumination and withdrawal. Further, previous day’s T2 stress and burnout did not significantly predict next day’s T3 rumination and withdrawal, even when controlling for previous T3 reports of coping. - At higher levels of spouses’ withdrawal, the association between paramedics’ rumination and paramedics’ T3 marital tension appeared to be exacerbated. Paramedics’ rumination also appeared to exacerbate spouses’ T3 marital tension when spouses’ withdrawal was high. No relationship was evident between paramedics’ rumination and spouses’ T3 marital tension at low spouse withdrawal. |  | 5 |
| **McCoy et al., 2009.**  USA  (39) | Police/ Law enforcement personnel vs general population. | Spouse | Cross-sectional survey data | 954,615 | Transit and railroad police n= 1143; detectives n= 122125; supervisors n= 129970;  Police officers n= 675756;  animal control officers n= 14051;  fish and game wardens n=3885;  parking enforcement n= 7685;  correction n= 492542;  officers n= 437616;  supervisors n= 54926. | **Divorce rates amongst law enforcement officers.** | Not reported | **2000 U.S. Census** using a special program developed by the Census Bureau, Data Ferret, which can be downloaded at http://dataferrett. census.gov/dataferrettapplicationinstall.exe.  2000 People and Housing One Percent Sample Census Survey. This dataset contained both a detailed breakdown of occupations as well as employees’ current (2000) marital status. The census collects data on 449 occupations. These occupations are divided into 23 major groups, 96 minor groups and 449 broad occupations. For police officers, the major group is Protective Service Occupations (33), the minor group is Law Enforcement Workers (33-3000), and the broad occupation is Police Officers (33-3050).  To compute the divorce rate for each occupation, they used the following formula: (Separated + Divorced) / (Total population – Never Married). | **Divorce/ separation rates/ results:**   - The divorce/ separation rates for law enforcement occupations (14.47%) was lower than both the national average (16.96%) as well as the rate expected given the demographic and income characteristics of the law enforcement workers (16.35%). - When law enforcement workers are broken down into broad occupations, the divorce rates for police officers (15.01%), supervisors (12.75%), detectives (12.53%), and railroad transit police (5.26%) are lower than the national average as well as the expected rate after controlling for demographics. However, the rates for animal control officers (19.02%), fish and game wardens (25.53%), and parking enforcement officers (26.25%) are higher than the national average as well as the demographically controlled expected rate. | **Correlational analyses:** Divorce rates higher for occupations with higher percentages of African Americans (r=.49) and women (r=.26), lower for occupations with higher percentages of Asian Americans (r= -.14), and lower for occupations with higher average incomes (r= -.53). |  | 2 |
| **McKeon et al., 2020**  Australia  (38) | Fire, police, ambulance | Informal caregiver | Cross-sectional | N=30 informal caregivers and N=34 Emergency Service Workers (ESWs)  N=64 in total), were included in this study. | The majority of informal carers (77%) were female  N =22 (73%) were life partners to the ESW, n=5 (17%) were close friends and n=3 (10%) were other family members.  The participants were informal carers of n=15 (44%) firefighters, n=13 (38%) ambulance and n= 6 (18%) police. | **Self-Reported Physical and Mental Health of Informal Caregivers of Emergency Service Workers** | N/A | **Kessler-10 (K10**) - 10 items scored on a five-point Likert scale with total scores ranging from 10 to 50. Scores were grouped into 4 levels of psychological distress; a score of 10–15 indicates low, 16–21 moderate, 22–29 high and 30–50 very high based  **The 21-item Depression Anxiety and Stress Scale (DASS-21**) was used to assess mental health symptoms (Henry & Crawford, 2005). A total score and three separate subscales, each with 7 items, were calculated to identify severity ratings for depression, anxiety and stress.  **The Pittsburgh sleep quality index** was used to assess participants quality and patterns of sleep in the past month (Buysse et al., 1989). Seven sub- scores were calculated ranging from 0 to 3 to yield a global score that can range from 0 to 21.  **The Assessment of Quality of Life-6D scale (AQoL-6D)** was used to assess the quality of life (Richardson et al., 2012). A total simple psychometric score for health-related quality of life and profile scores on the different dimensions were calculated. Scores can range from 20 to 99 with higher numbers representing better quality of life.  **The Simple Physical Activity Questionnaire (SIMPAQ)** is a 5-item clinical tool designed to assess physical activity among populations at high risk of sedentary behaviour (Rosenbaum et al., 2020) | High rates of psychological and physical morbidity were observed among the informal caregivers of ESWs.  N.b small sample size limitations in generalisability | Psychological distress   - Among the informal caregivers, 67% (n=20) were experiencing moderate to very high levels of psychological distress, with nearly one-third of participants (n=9, 30%) experiencing very high levels of distress. Higher than general population reporting 13% high levels of distress. - Mean scores as assessed by the DASS-21 for the informal caregivers were 4.8 (SD 4.5) for depression, 3.9 (SD 3.7) for anxiety and 7.2 (SD 5.3) for stress. These mean scores were categorized as mild depression, mild anxiety and normal stress (Crawford et al., 2011). Scores on each of the DASS-21 subscales were worse than the Australian population norms. - The informal caregivers mean PSQI scores were 8.8 (SD 4.0), indicating worse sleep than the Australia general population mean of 6.3 (SD 3.4) - The informal carers were also experiencing poor quality of life 79.1 (SD 10.8), compared to the general population mean of 84.37 (SD 11.5). The emergency service workers were experiencing lower quality of life than the general population, however, no statistical difference was found between the informal caregivers and ESWs. - The informal carers mean sedentary time was 13.2 (SD 4.5) hours per day. The informal caregivers were less active than the general population, with only 30% of the participants meeting the physical activity guidelines of 150 min of moderate to vigorous activity per week compared to 55% in the general population |  | 4 |
| **Meffert et al., 2014.**  United States  (36) | Police officers: from training to commencing work | Spouses/ partners | Longitudinal: repeated measures questionnaire design | 71 police recruits + 71 Spouses/partner (S/Ps) | Police recruits: Male (n=71) = 92%  S/Ps: female (n=62) = 94%.  50% of police recruits and S/Ps were white. 10–20% were black, Hispanic or Asian in both recruits and S/Ps.  For both recruits and S/Ps, bachelor’s degree/completed 4 years of undergraduate study = 46% in police; 40% in S/Ps. | **PTSD and spouses/ partners emotional distress, and relationship violence:** | Not reported | **Dependent Variables**:  ***Modified Secondary Traumatic Stress Questionnaire (MSTQ):*** 28-item Secondary Trauma Scale (STS): 5-point Likert scale - scores of 45 or higher indicative of problematic symptoms of intrusion and avoidance that should be of substantive clinical concern. Cronbach’s alpha = .92.  ***Conflict Tactics Scale (CTS)***: *Straus et al., 1996: The Revised Conflict Tactics Scales (CTS2).*  **Independent Variables:**  ***Beck Depression Inventory (BDI)***: *Beck et al 1961: An inventory for measuring depression:* The Second Edition (BDI-II) is a 21-item assessment of depression symptoms over the past 2 weeks. 4-point scale. Cronbach alpha =0.861.  ***Critical Incident History Questionnaire (CIHQ)***: *Weiss et al., 2010:* *Frequency and severity approaches to indexing exposure to trauma:* *The Critical Incident History Questionnaire for police officers:*34-items. Total cumulative exposure score is derived by summing the frequency of incident exposure across all items. Obtained from officers at 12months and a modified version from S/Ps.  ***Mississippi Combat Scale-Civilian Version (MCS-CV)***: *Keane et al., 1988:* *Mississippi Scale for Combat Related Posttraumatic Stress Disorder:* 35-item measure that assesses PTSD-related symptoms of intrusion, avoidance, hyperarousal, and other difficulties since the time of critical incident or trauma exposure.  5- point Likert scale. Items 2, 6, 11, 17, 19, 22, 24, 27, 30, and 34 are reverse coded. Items are summed to obtain the total MCS-CV score.  Police officers reported symptoms since beginning police service & S/Ps reported symptoms of the officers with whom they were in a relationship.  ***Symptom Checklist-90-R (SCL-90-R):*** *Derogatis LR, Lipman RS, Covi L (1973) SCL-90:* Used to evaluate a broad range of psychological problems and symptoms of psychopathology (4-point likert scale).  **Baseline Assessment and follow up assessment at 12 months**  *STSS is referred to as PTSD for the purpose of this study. | - S/P perception of PTSD symptoms may play a key role in the spread of traumatic stress symptoms across intimate partner relationships and intimate partner violence in the context of PTSD. - Average S/P secondary traumatic stress scores were relatively low at 12 months (26.8), corresponding to mild symptoms. - S/P perception of officer PTSD symptoms at 12 months was significantly associated with S/ P secondary traumatic stress and couple violence. - There was no significant correlation between officer report of PTSD symptoms and S/P secondary trauma. | **Baseline:**   - S/Ps reported low levels of depression and hostility and approximately 1/4 reported couple violence. - At 12 months, the average S/P STS score was 26.8, corresponding to mild symptoms and 5 S/Ps (7%) met the threshold for secondary trauma on the MSTQ. - The average officer MCS-CV symptom score at 12 months (59.82) was below that of PTSD patients and psychiatric populations (86). No officers met criteria for full PTSD disorder at 12 months.   **Correlations:**   - Total couple violence and S/Ps’ violence toward officers were significantly correlated with S/P baseline hostility (r = .28, .31, respectively), baseline violence (r = .78, .75), S/P report of officer PTSD at 12 months (Pearson’s rho = .33, .33) and S/P secondary trauma at 12 months (r = .33, .38).   **Predictors of STS among S/Ps of police officers:**   - S/P education, baseline depression symptoms and reports of officer’s PTSD symptoms at 12 months were significantly and directly associated with secondary traumatic stress among S/Ps at 12 months. The final model accounted for 44.7% of the variance in STS among S/Ps (adjusted R squared).   **Predictors of Violence:**   - Nested logistic regression was used to evaluate the predictors of total couple violence and S/P to officer violence. In both models after entering baseline violence, none of the covariates were significant at any step until S/P perception of officer PTSD symptoms was added in the final step. In the two final models both baseline violence and S/P perception of officer PTSD were significant predictors of S/P to officer violence. | **They observed a discrepancy in report of PTSD symptoms with officers reporting higher PTSD symptoms than perceived by their S/Ps.** The S/P-officer PTSD symptom discrepancy found in this study may partly explain why S/P distress was not associated with officer PTSD.  **Significant predictors of both total couple violence and S/P to officer violence included baseline violence and S/P perception of officer PTSD symptoms**. | 5 |
| **Pfefferbaum et al., 2002.**  United States  (68) | Firefighters | Spouses | Cross-sectional questionnaire | 27 partners of Oklahoma City firefighters who served in the rescue and recovery following the Oklahoma City bombing. | 100% = women | **Mental health:**  spouses and intimate partners of Oklahoma City firefighters who participated in the rescue/ vicarious trauma exposure. | Not Reported | **The Diagnostic Interview Schedule (DIS) for the Diagnostic and Statistical Manual of Mental Disorders, Revised Third Edition (**DSM-II Robins LN, Helzer JE, Cottler L, et al. NIMH Diagnostic Interview Schedule, Version 3–Revised. St. Louis, MO: Washington University; 1989I-R); American Psychiatric Association. Diagnostic and Statistical Manual of Mental Disorders, 3rd ed., revised. Washington, DC: APA; 1987.  **Onset and recency of symptoms were also documented to ascertain lifetime and current diagnoses.**  **The disaster supplement:** Robins LN, Smith EM. The Diagnostic Interview Schedule/Disaster Supplement. St. Louis, MO: Washington University; 1983: information about participant demographics; disaster experience, including exposure to the event, perceptions of terror and horror, other subjective impressions, and physical injuries; level of functioning; and mental health treatment.  Perceptions of their partners reactions to the bombing, the **degree to which their partner had been affected by the incident** and the **degree to which he had recovered and establishing the extent of psychiatric symptomatology in their mate and its impact on functioning**.  **A checklist of posttraumatic stress and depressive symptoms** and asked to indicate whether her partner had experienced those symptoms after the disaster and, if so, to indicate whether they were new symptoms afterward or had been present prior to the disaster. Participants were also asked about excessive use of alcohol by partner. | - Low rates of post disaster disorders, and most of this psychopathology pre-existed the bombing. - 1 (4%) woman in the study developed bomb-related PTSD, and this woman also had major depression. This is considerably lower (χ2 = 10.4, df = 1, P < .002) than the 13% rate of developing PTSD in the firefighters. - Some of the partners reported changes in their health, functioning, and relationships. While almost 50% of the sample acknowledged changes in relationships with their partners, these changes were no more often negative than positive. Relatively few women described problems in their relationships with their firefighter mate as a result of the bombing, and none reported bomb-related problems in other relationships. - These women believed the bombing to be both upsetting and harmful to their firefighter mates and described high rates of depressive symptoms in them. 1 (4%) woman reported excessive alcohol use in her partner. - Most of these women coped by turning to family and friends. This suggests that efforts to support the firefighters should include attention to their interpersonal support networks, such as families and friends. This is especially important given their apparently infrequent utilization of formal mental health services. - 2 (7%) of the women in this sample sought professional attention. | **Demographics:**   - Predominantly Anglo, in their late thirties, and had been married to or cohabitating with their firefighter partner for about 10 years. Most heard and felt the bomb blast, but none were injured. The majority were secondarily exposed by knowing others directly exposed to and/ or killed by the bombing   **Mental Health:**   - The lifetime rate of psychiatric disorders in the 27 women prior to the bombing was 26% (n = 7), with major depression in 7 (26%), panic disorder in 1 (4%), and generalized anxiety disorder in 1 (4%). - The post bombing prevalence of psychiatric disorders was 22% (n = 6), with PTSD in 1 (4%), major depression in 5 (19%), and panic disorder in 1 (4%). The woman with PTSD also had major depression after the bombing. Of the 6 women with a post disaster disorder, 5 met criteria for a disorder predating the bombing, and 5 of the 7 women with a pre disaster disorder met criteria for a post disaster disorder. The 1 woman with a new disorder after the bombing developed both PTSD and major depression. The 2 cases of pre disaster disorders that were nonrecurrent after the bombing occurred in 2 women with pre-existing major depression. - More than 1/2 (n = 14, 52%) of the women reported having one or more bombing-related PTSD symptom with a mean (SD) of 2.4 symptoms. There were 12 (44%) women who met PTSD group B (intrusive reexperiencing) criteria, 1 (4%) met PTSD symptom group C (avoidance/ numbing) criteria, and 9 (33%) met group D (hyperarousal) criteria after the bombing. - Before the bombing, 8 (30%) of the women had consulted a mental health professional, and another 7 (26%) had elected not to seek professional help at a time when they thought they needed it.   **Impact on Functioning, Health, Relationships, and Perceptions:**   - Most (n = 18, 66%) of the women indicated that, in the last month, they were satisfied “all” or “almost all” the time with their job performance; 2 (7%) were satisfied “a good deal” of the time, and 1 (4%) “not much” of the time. Satisfaction with job performance was not related to presence or absence of a psychiatric diagnosis, meeting PTSD symptom group criteria, or number of PTSD symptoms. - A minority (n = 6, 22%) of the women said the bombing affected their job satisfaction; for 2 (7%), it was affected in a positive direction, and for 4 (15%), it was in a negative direction. Job satisfaction was not related to presence or absence of a psychiatric diagnosis or with meeting PTSD symptom group criteria. - A higher number of PTSD symptoms was associated with change in job satisfaction for the worse (6.8 [SD = 2.2] vs. 1.7 [SD = 2.3]; t = 4.04, df = 25, P < .001). Specifically, worsened job satisfaction was associated with the number of hyperarousal (PTSD group D) symptoms (3.3 [SD = 1.3] vs. 0.8 [SD = 1.2]; t = 3.73, df = 25, P < .001), but not with the other two PTSD symptom groups. - 4 (15%) women reported their health had worsened since the bombing; 3 (11%) said their health had improved. Changes in health were not related to presence or absence of a psychiatric diagnosis, meeting PTSD symptom group criteria, or number of PTSD symptoms. - 1/3 (n = 10, 37%) of the women indicated that they had experienced some permanent change in their interpersonal relationships as a result of the bombing, and another 2 (7%) reported changes in relationships that were only temporary. - 1/2 (n = 13, 48%), noticed changes in their relationship with their firefighter mate. Specifically, communication in the relationship changed for the positive in 5 (19%) and for the negative in 2 (7%), intimacy was improved in 3 (11%). Shared goals were improved in 4 (15%) and changed for the negative in 1 (4%), sexual relations improved in 2 (7%) and shared parenting worsened in 2 (7%). - 1/4 (n = 7, 26%) said they personally had been affected “very much” by the bombing, 18 (67%) “somewhat,” 2 (7%) were affected “very little.” - Most (n = 23, 85%) of the women felt the disaster was very upsetting, and 4 (15%) felt it somewhat upsetting. - Most (n = 19, 70%) of the firefighter partners reported no change in frequency of church attendance, but 3 (11%) said they were attending more since the bombing, and 5 (19%) were attending less, 8 (30%) said the bombing had affected their religious beliefs; 7 of these said it had strengthened their beliefs, and 1 said she started to question her beliefs.   **Coping:**   - The majority (n = 17, 63%) coped by turning to friends or relatives, and 8 (30%) felt this was the most helpful coping strategy they used. | **Perception of Firefighter Partner Response**  8 (30%) women felt the bombing had caused their firefighter partner a great deal of harm. Most (n = 22, 81%) described the bombing as very upsetting for their partner, 5 (19%) as somewhat upsetting. 8 (30%) felt their partner had been very affected by the bombing, another 12 (44%) felt the partner was somewhat affected, and 7 (26%) felt he was affected very little; none reported no effect.  1/2 (n = 14, 52%) said their partner had fully recovered from the disaster, 12 (44%) felt he had partially recovered, and 1 (4%) felt he had not recovered. Many (n = 18, 67%) of the firefighters were sad or depressed, and only 2 (7%) indicated this predated the bombing. Sleep disturbance was also common (n = 17, 63%), although it predated the bombing in some (n = 6, 22%). 4 (15%) stated their partners had flashbacks. 1 (4%) woman reported that her partner was using too much alcohol.  1/3 (n = 9, 33%) of the women said they and their partners talked about the bombing “rarely” or “not at all,” 7 (26%) talked about it “sometimes,” 10 (37%) talked about it “frequently,” and 1 (4%) talked about it “too much.” | 3 |
| **Richardson et al. 2016**  United States  (69) | Firefighters | Spouses – specifically widows from 9/11 | Online Survey | N=55 |  | **Social support and Post Traumatic Growth (PTG)** | - | **Post Traumatic Growth Inventory** (Tedeschi & Calhoun 1996)  Social support used and perceived quality of social support services | - Importance of formal one to one counselling however priority importance of informal peer support in aftermath of in the line of duty deaths. - Informal support played a lasting an enduring role in aiding widows’ recoveries over 10 years post 9/11 incident - Higher levels of PTG experienced by FDNY 9/11 widows compared to other groups. - Formal and informal support correlated with PTG - Overall evidence suggesting widows rebuilding lives in aftermath of husband’s loss and embracing change supported by informal networks of support. | **Social Support**:   - 50% one to one counselling in first two years after attacks, 40% 2003 slow decline to 30% in 2011. - 55% reported one to one counselling had assisted with personal healing to a ‘great’ or ‘very great’ extent. - Formal group therapy dramatic decline in 2011 to under 10% - Most frequent form of social support was meeting informally with other 9/11 widows 70% meeting 2001-2002 specifically related to 9/11 and 73% to socialise. By 2011 60% still meeting other widows to socialise. - 58% reported meeting informally with other widows had assisted with their personal healing to a ‘great’ or ‘very great’ extent.   **Post Traumatic Growth:**   - Average PTG score 65.3 (range 0-105) (slightly higher than average score reported by Baker et al. 2008 of 50) - PTG was positively correlated with:   - Fire Department New York (FDNY)-sponsored support groups (r = .37, p < .01)  - one-on-one therapy (r = .37, p < .01)  - socializing with other FDNY widows (r = .29, p < .05)  - local support group (r = .27, p < .05).   - Examination of the PTG subscales revealed that “relate to others” was positively correlated with one-on-one therapy (r = .42, p = .001) and FDNY support groups (r = .31, p < .01). - “New possibilities” was positively correlated with local support groups (r = .27, p < .05) and FDNY support groups (r = .27, p < .05). - “Personal strength” was related to FDNY support groups (r = .27, p < .05). - “Spiritual change” was positively correlated with FDNY support groups (r = .45, p ¼ .001) and discussing 9/11 with other FDNY widows (r = .39, p < .01). - “Appreciation for life” was positively correlated with socializing with other FDNY widows (r = .36, p < .01) and one-on-one therapy (r= .29, p < .05). | Importance of finding quality interaction with the agency where the spouse works when there is a line of duty death.  Need for organisations to reach out to newly widowed individuals and put them in touch with those who have been there before. | 4 |
| **Roberts 2001**  United States  (49) | Police | Spouse and police partner data | Questionnaire data, daily stress diaries and laboratory interaction sessions | 19 |  | **Impact of job stress and physical exhaustion on marital interaction** | - | Spouses questionnaires: **demographics**  **marital satisfaction** (Locke & Wallace, 1959),  **areas of disagreement in the couple’s relationship** (Gottman, Markman, & Notarius, 1977),  **health (the Symptom Checklist-53,** Derogatis & Lazarus,1994; the Wahler Symptom Checklist, Wahler,1968)  **recent life stress** (Horowitz, Schaefer,Hiroto, Wilner, & Levin, 1977).  Officers completed the **Police Stress Survey** (Spielberger,Westberry, Grier, & Greenfield, 1981), which assesses the extent to which officers perceive 60 job-related events as stressful (using a 0–100scale). Officers’ ratings on the Police Stress Survey were totalled, and the average rating of perceived stress was calculated.  Over a 30-day period, officers and their spouses **completed daily stress diaries** using a 9 point likert scale to rate the following as it applied to that day: (a) how much stress they experienced related to their job, (b) how much pleasure they experienced related to their job, (c)how much stress they experienced related to their marriage, (d) how much pleasure they experienced related to their marriage, (e) how many hours they worked, and (f) how many hours they spent with their spouse.  **Interaction sessions:** Couples were recorded discussing their day and physiological measures were taken to detect participants positive and negative emotional states. Couples re-watched their interaction and self-rated how they felt second by second at the time using an interactive dial (extremely negative to extremely positive on a 9-point likert scale) | - Officers carried their job stress home that influenced their interactions with their wives. - Influences of job stress were found regardless of marital satisfaction, shift work and parenthood status. - Job stress deemed more toxic for marital interaction than physical exhaustion. A husband’s job stress produces a climate in which both spouses show signs associated with future marital distress and dissolution (heightened cardiovascular arousal, increased negative affect, decreased positive affect, greater emotional distance and disconnectedness) - Found evidence on high stress days of individuals trying to regulate their emotions. - Additionally, husbands on high stress days displaying vigilance and defensiveness may find their spouses assume similar postures in anticipation of martial interactions of efforts to avoid exacerbating their husbands’ stress - Job stress dampens positive marital interaction. - Wives may attempt to improve emotional climate on high stress days and not exacerbate stress, however in the long term this may create distance of spouses to husbands - Physical exhaustion in husbands was not reflected in physical exhaustion of their spouses – therefore physical exhaustion did not produce charged and dangerous marital climate in the way job stress did. | Complex statistics – recommend referring to original paper | Couples need to be attuned to the days when working spouses have experienced high levels of job stress and find ways to manage this constructively – i.e., stress management techniques, finding constructive ways to talk about job stress rather than avoid it.  Job stress can have serious implications for families and successful marriages | 3 |
| **Roberts 2013**  United States  (47) | Police | Data from police officers and spouses | Questionnaires, daily diaries, 4x laboratory sessions | 17 Police officers and 17 spouses |  | Effects of stress on police marriages |  | **Marital Satisfaction (officer and spouse)** (15-item Locke-Wallace Marital Adjustment test, Lock and Wallace 1959)  **Police Stress** (Officers) (Spielberger 1981 Police Stress Survey)  **Daily diaries** (Officers and spouses)  Using a 9-point Likert scale participants rated how much stress and pleasure they experienced from the job and marriage each day, numbers of hours worked and with spouse.  **Emotional behaviour** (Waldinger 2004)**:** Five coders watched videotapes of each couples 5-minute silent period and 15-minute conversation, focusing on one partner at a time. After every 30 seconds, coders rated how much the target participant was demonstrating 22 behaviours from a prior list. Inter-rater reliability assessed. | - When officers reported more job stress, they showed less hostility, less synchrony with their wives’ hostility, and more synchrony with their wives’ affection; their wives showed greater synchrony with officers’ hostility and less synchrony with officers’ affection. - Therefore, for officers, greater job stress was associated with less behavioural negativity, potentially less attunement to wives’ negativity, but potentially greater attunement to wives’ affection—perhaps a compensatory strategy or attempt to buffer their marriage from stress. These attempts may be less effective, however, if, as the synchrony findings may suggest, wives are focusing on officers’ hostility rather than affection. | - Based on daily diaries, officers reported greater job pleasure, t(10) = 2.59, p < .03, and marital pleasure, t(12) = 2.55, p < .03, than their wives. - Officers and wives did not differ in amount of job stress, marital stress, or marital satisfaction reported. - During couples’ conversations, both officers and their wives displayed more affection behaviours than hostility behaviours, t(154) = 2.32, p < .03 (officers), and t(154) = 6.81, p < .001 (wives). Wives showed greater mean levels of affection behaviours than officers, t(1341) = 2.34, p < .02; officers and wives did not differ in mean levels of hostility. - For wives, higher global marital satisfaction (Locke–Wallace) was associated with more affection, t(1339) = 2.36, p < .02, and less hostility, t(1339) = 3.53, p < .001. Similarly, greater marital pleasure (diary reports) was associated with less hostility for both officers, t(1023) = 2.21, p < .03, and wives, t(1023) = 1.98, p < .05. Finally, greater marital stress (diary reports) was associated with more hostility for wives, t(1023) = 2.12, p < .04. - Found that strength of association was stronger for officers’ behaviour predicting wives than the other way around (sex difference for hostility: b = 0.37, t[1339] = 8.28, p < .001; sex difference for affection: b = 0.18, t[1339] = 4.21, p < .001). Such asymmetry can be interpreted as meaning that variance in wives’ behaviour is more fully accounted for by their husbands’ behaviours than the other way around.   **Officer Stress and Mean Levels of Couple Emotional Behaviour:**   - Greater stress predicted lower mean levels of officer hostility behaviours during couple interactions, t(1023) = 2.63, p < .002 (police stress past month), and t(1023) = 2.08, p < .04 (perceptions of police work as stressful). Officers’ reports of stress did not predict wives’ hostility behaviours during couples’ interactions. - **Affection:** Officer stress did not significantly predict mean levels of affection behaviours for officers or their wives. - **Hostility synchrony:** Officers reporting more stress showed less synchrony with their wives’ hostility behaviours, t(860) = 5.01, p < .001 (stress past week), and t(1019) = 2.63, p < .01 (stress past month). In other words, when officers reported more recent stress, officers’ hostility was less strongly predicted by their wives’ hostility. - In contrast, for one measure of officer stress—police stress the past year—greater officer stress predicted more hostility synchrony for wives, t(1019) = 2.49, p < .02. In other words, when officers reported more stress the past year, their wives’ hostility was more strongly predicted by officers’ hostility. - **Affection Synchronicity:** For three of four measures of officer stress, greater stress was associated with less affection synchrony for wives (i.e., wives’ affection was less strongly predicted by officers’ affection): stress past month: t(1019) = 3.70, p < .001; stress past year: t(1019) = 3.33, p < .001; perceptions of stress: t(1019) = 2.24, p < .03. - Officers reporting more stress showed more synchrony of affection (i.e., officers’ affection was more strongly predicted by their wives’ affection) for three of four stress measures: past week: t(860) = 1.99, p < .05; past month: t(1019) = 4.31, p < .001; past year: t(1019) = 2.42, p < .02. |  | 4 |
| **Ryan et al., 2000**  United States  (52) | Police | Data from Police officers on domestic violence (DV) | Questionnaires | 210 |  | **Domestic Violence** | 73% | **Questions on involvement of officers in domestic violence, discipline of perpetrators, training, degree to which domestic violence exists as a problem in their department.**  **Personal History Questionnaire** (One question asked, “Have you ever slapped, punched, or otherwise injured a spouse or roman- tic partner?”) | - Very low pre-hire estimate of those involved in domestic violence, therefore hypotheses that domestic violence may be as a result of the policing profession. - This study not able to give a representative prevalence of domestic violence. | - 54% indicated they knew an officer in their department involved in domestic violence. - 64% stated they had heard rumours about officers involved in domestic violence. - 44% stated domestic violence occurred among members of their department. - 45% acknowledged reports to their departments concerning domestic violence by officers. Only 16% knew of officers involved in domestic violence that went unreported. - 31% reported that a member of their department had been disciplined for involvement in domestic violence. 28% stated they believed severe punishment caused underreporting of domestic violence. 30% stated they believed punishments to be too severe. 63% stated that officers would seek help if the information remained confidential. - 10% of respondents admitted to ever having slapped, punched or otherwise injured a spouse or partner – 2% indicated this had happened 2-3 times |  | 3 |
| **Sanford et al. 2017**  United States  (48) | Firefighters | Relationship data from First Responder (FR) | Online Questionnaire | 102 |  | **Testing Couple Resiliency Inventory** | - | **Couple Resiliency Inventory** (Sandford et al. 2015) – includes 9 positive and 9 negative behaviours in relationships. E.g., ‘one partner helped the other by maintaining a positive attitude and being optimistic, ‘either you or your partner was attentive to the other’s needs,’ ‘either you or your partner withdrew from communication,’ and ‘either you or your partner was critical, or hostile, or blamed the other’  **WHO Wellbeing Index** (Bech 2004) includes five items that assess a person’s overall perception of wellbe- ing, and thereby an absence of depression or anxiety. A sample item is, ‘I feel cheerful and in good spirits.’  **Perceived Job Stress** (Kimbrel et al. 2011 – 14 items) Participants rated the extent to which they were ‘bothered by’ 14 different sources of job-related stress that are sometimes experienced by firefighters and paramedics (such as having a poor diet, thoughts about past runs that have been upsetting.)  **Exposure to traumatic events** (11-items from International Association of Fire Fighters 2001)  **Relationship satisfaction (**Funk & Rogge 2007 – 4 items) | - Clear distinction between relationship satisfaction and couple resiliency. - Positive and negative resiliency had direct effects explaining unique variance in general wellbeing. - Levels of job stress were significantly predicted only by negative resiliency. - Relationship satisfaction partially mediated effects of both positive and negative resilience | - Positive and negative resiliency had direct effects explaining unique variance in general wellbeing. (0.21, p=0.05 , 0.37, p=0.05) - Levels of job stress were significantly predicted only by negative resiliency (0.45, p=0.05) - Relationship satisfaction partially mediated effects of both positive and negative resilience (Standardised indirect effect= 0.11, p=0.05, standardised indirect effect-0.13, p=0.05) |  | 4 |
| **Shakespeare-Finch et al. 2002**  Australia  (66) | Ambulance | First Responder data on family functioning | Questionnaire | 71 | Ambulance officers n=39  Control group (gen public) n=32 | **Trauma, coping and family functioning** | Ambulance = 89% control group = 94% | **Family Functioning – Intimacy Conflict Parenting Styles (ICPS – Noller et al. 1992)** The ICPS scales are incorporated into a 30-item questionnaire designed to measure three dimensions of family functioning: Intimacy (high vs. low), Conflict (high vs. low), and Parenting style (democratic vs. controlling) (Noller *et al*., 1992). Responses were measured on a 6-point Likert-type scale ranging from 1= totally disagree to 6= totally agree.  **Coping Resources – Personal Resources Questionnaire (PRQ) (adapted Neale 1991)** The PRQ is intended to measure the capacity to cope effectively with occupational stress. The PRQ consists of four subscales, each containing 10 items that measure the extent to which participants utilize the coping resources of recreation, self-care, social support, and rational cognitions | - Personal resources found to have a significant impact on dimension of family functioning. - Ambulance officers demonstrated a more varied repertoire of personal resources than the control group. - Authors suggest ambulance officers may have the ability to compartmentalise their experiences effectively so that traumatic incidents in the workplace do not spill over into family environment. - Possible that previous traumatic experiences enabled the ambulance group to cope more effectively with a broader range of techniques from an ‘experiential learning effect’. | - In ambulance group, social support was predictive of intimacy levels. (β=0.54, p=0.003) - Rational-cognitive strategies were predictive of conflict levels (β=- 0.46, p=0.003) - Self-care, social support and rational cognitive strategies were all significantly correlated with three dimensions of family functioning |  | 5 |
| **Thompson et al., 2005**  Australia  (50) | Police | Family Functioning | Questionnaire | 421 |  | **Work stress and family functioning** | 42% | **Social Support** (6-items Ray and Miller 1994) Each scale had six items scored on a five point Likert scale from strongly disagree (1) to strongly agree (5). Ray and Miller (1994) reported the Supervisor Support scale to have a reliability of a = 0.92 and the Coworker Support scale to have a reliability of a = 0.90. Sample items are ‘My supervisor respects me’ and ‘My coworkers can be relied on when things get tough at work’.  **Work Stress** – occupational stress Inventory – Role ambiguity and role overload (Osipow and Spokane 1981) These scales contain 10 items each, answered on a five point Likert scale from rarely or never true (1) to true most of the time (5). An example item is ‘I am expected to do more than is reasonable’ (Role Overload) and ‘My supervisor asks for one thing but really wants another’ (Role Ambiguity).  **Emotional Exhaustion** – adapted emotional exhaustion scale (Maslach and Jackson 1981) Each item is scored on a five point Likert scale from strongly disagree (1) to strongly agree (5). An example item was ‘I feel used up at the end of the workday’  **Family environment.** Family environment was measured using two scales from the Family Environment Scale (Moos & Moos, 1994). To measure conflict in the family the Family Conflict Scale (internal reliability (Cronbach’s a) of 0.75) was used, and to measure the degree of felt togetherness in the family, the Family Cohesion Scale (internal reliability (Cronbach’s Alpha) of 0.78) was used. Each scale had 10 items scored on a five point Likert scale from strongly disagree (1) to strongly agree (5). Sample items are ‘Family members really help and support each other’ (Family Cohesion) and ‘We fight a lot in our family’ (Family Conflict). | - The policewomen in this study reported spillover effects of work stress to their family environment in terms of levels of emotional exhaustion, and perceptions of reduced family cohesion, but also reduced family conflict, an unexpected finding. In particular, work stress impacted on the family environment through emotional exhaustion, a component of burnout. - Supervisor support reduced work stressors of role overload and role ambiguity, and consequently emotional exhaustion. Thus supervisor support is associated with perceptions of the family environment, via its impact on role stressors and emotional exhaustion - The finding that emotional exhaustion mediated the effect of work role stressors on the family environment supports suggestions that mood may be a mechanism by which work stress affects the family environment - Perceptions of lower levels of family conflict with increased emotional exhaustion were unexpected but may reflect several possible mechanisms. It is notable that with increased stress reports of withdrawal from interaction in the family occur and consequently to some extent these women may avoid interactions leading to conflict when they are emotionally exhausted as a way of conserving resources. | - Significant negative pathway between supervisor support and both role overload (b = -0.22) and role ambiguity (b = -0.42). - Co-worker support was not significantly associated with either role overload or role ambiguity. - Both role overload (b = 0.46) and role ambiguity (b = 0.17) were significantly and positively associated with emotional exhaustion. - Finally, emotional exhaustion was significantly and negatively associated with both family conflict (b = -0.12) and family cohesion (b = -0.12) although the proportion of variance explained in both cases (2 per cent) was small. | A fruitful avenue of exploration of stress transmission to the family would be an examination of behaviours linked to emotional exhaustion. Additionally, interventions designed to reduce stress in policewomen should include supervisor training in social support | 4 |
| **Uchida et al., 2018**  United States  (63) | Police | Responder Parent and Child data | Questionnaires | 16,386 | World Trade Centre Responders  n=8034 police and n=8352 non-traditional (e.g., construction workers)—with one or more children | **Trans-generational associations between Post Traumatic Stress Disorder (PTSD) symptoms in World Trade Center (WTC) responders and behavioural problems in their children** |  | A questionnaire from the **Disaster Supplement of the Diagnostic Interview Schedule**,27 with responders as informants, assessed 12 child behavioural problems evident during two time periods; during the time that the responder was working at the WTC site and in the month prior to the worker's first visit to the WTC-HP.  **Total number of WTC exposures**  **Social support -** Sources of family and work social support were assessed by asking respondents to endorse important sources of family and work support while working on the WTC effort. Responses were used to create a dichotomous variable of work-related support (no support vs support from boss and/or co-workers) and a count variable of number of family-related supports (ie, from spouse, partner, child(ren), parent(s), and/or other family).  **WTC-related medical conditions**  **Pre 9/11 psychiatric diagnosis** – participants asked if they were diagnosed with depression/anxiety/PTSD prior to 9/11  **Stressful life events since 9/11** – derived from Disaster Supplement of the diagnostic Interview Schedule (Robins 1983)  **WTC-related PTSD symptoms – PCL-S (Weathers 1993)** | - Crucial symptomology of police responders which impacts their child’s behavioural difficulties. - Parental dysphoric arousal symptoms, which are characterized by anger/irritability, sleep disturbance, and concentration difficulties, were significantly associated with all domains of child behavioural problems assessed, including fearful/clingy, externalizing behaviours, and somatic problems. - A total of 31.4% of non-traditional and 20.0% of police responders reported behavioural problems in their children. Non-traditional responder status, female sex, Hispanic ethnicity, more life stressors, more WTC-related PTSD symptoms, and dysphoric arousal symptoms were significant correlates of behavioural problems in responders’ children. - Specific parental sociodemographic, psychosocial, and clinical characteristics, as well as PTSD symptom severity, were significant correlates of child behaviour problems. - Differences in police and non-traditional responders may be: 1) lack of disaster response training in non-traditional responders, increasing PTSD prevalence and affecting children, 2) children of police may be more accustomed to having a parent in high stress occupation, 3) police children may have already had behavioural problems pre 9/11 and therefore police parents note less change 4) police parents may underreport children’s behaviours as they underreport their own mental health. | - Police and non-traditional responders differed significantly on all demographic, exposure, and clinical characteristics (*P* < 0.5). Notably, police responders were more likely than non-traditional responders to be white/non-Hispanic, have continued schooling beyond high school, report substantially lower prevalence of previous psychiatric diagnoses, and report more social support at work. While police responders reported higher levels of WTC-related exposures, they reported fewer WTC-related PTSD symptoms than non-traditional responders and were less likely than non-traditional responders to have probable WTC-related PTSD (PCL-S score ≧ 44) - WTC-related PTSD symptoms – Police (M=25.4, SD=11.1), non-traditional responders (M=35.8, SD=16.6) - A total of 1608 (20.0%) of police responders and 2619 (31.4%) non-traditional responders with children under the age of 18 endorsed any child behavioural problem. - The most commonly endorsed type of child behavioural problem was fearful/clingy behaviour, followed by externalizing behaviour and somatic problems. Non-traditional responders were significantly more likely than police responders to endorse any of the child behaviour problems assessed, all χ2(1) > 41.43, all *P's* < 0.001. - In both police and non-traditional responders, female sex, Hispanic ethnicity, greater number of life stressors in the year before (Police AOR=1.09, p<0.01) and since (Police AOR=1.07,p<0.01) September 11, 2001, and higher WTC-related PTSD symptom levels (Police AOR=1.05, p<0.001) were associated with reporting any child behaviour problem. |  | 4 |
| **Zavala et al., 2013**  United States  (55) | Police | Spouse – domestic abuse – data from emergency responder | Questionnaire | 860 |  | **Effect of physical abuse on family violence** | 92% | **Single item questions asking whether any of their family got physical/violent with them when a child, whether they get physical with their spouse and children.** | - Police officers who indicated that their parents got physical with them were more likely to report being physical with their spouse and children. They were also likely to report yelling or shouting toward family members. In sum, this study shows a relationship between child maltreatment and involvement in family violence and increases the knowledge regarding the cycle of violence in police families | - 8.95% reported being violent towards their children. - 8.72% reported physical violence towards their spouse. - Police officers who reported that their parents got physical with them were almost 3 (odds ratio [OR] = 2.993; *p* ≤ .000) times more likely to physically abuse their spouses than respondents who did not report child maltreatment. - Officers who reported that their parents got physical with them were 4 times (OR = 4.149, *p* ≤ 000) more likely to report being physical with their children than officers who reported no child maltreatment.      - Police officers who had experienced physical maltreatment as a child were almost twice as likely (OR = 1.786, *p* ≤ .05) to yell or shout toward family members than officers who reported no child maltreatment. |  | 3 |
| **Zavala et al., 2015**  United States  (56) | Police | Spouse/ significant other but IPV data from ER | Cross-sectional analysis of already existing dataset- survey from 1997 | 1104 |  | **Influence of critical incident stressors, parental violence, and experiencing child maltreatment on Intimate Partner Violence (IPV) perpetration by police officers** | 93.2% | **Perpetration of IPV** (Anderson & Lo, 2011; Gibson, Swatt, & Jolicoeur, 2001; Zavala, 2013a). Respondents were asked the following question: “Have you ever gotten out of control and been physical (e.g., pushing, shoving, grabbing) with your spouse/significant other?” Respondents were allowed to answer either yes or no (0 = No, 1 = Yes)  ***Interparental violence*:** respondents were asked the following question: “Did your parents ever get physical with each other?” Respondents were allowed to answer either yes or no (0 = No, 1 = Yes).  ***Child maltreatment****:* “Have your parents (when you were a child) ever gotten physical with you?” Respondents were allowed to answer either yes or no (0 = No, 1 = Yes)  ***nonviolent values:*** measure was captured by asking respondents three questions about the appropriateness of violence in intimate relationships (e.g.,“A person who refuses to have sex with his or her spouse/significant other is asking to be beaten,” Respondents were allowed to answer these questions using a 5-point Likert scale (1 = strongly agree to 5 = strongly disagree). These three items were summed, with higher scores indicating higher levels of disapproval for violence (nonviolent values). A reliability analysis indicated an alpha level of .78.  ***critical incident stressors*:** Respondents were asked whether they have ever participated nine events such as: “making a violent arrest,” “shooting someone,” “being the subject of an internal investigation,” Respondents who experienced these events were further asked how much these events emotionally affected them, with response categories of “not at all,” “a little,” and “very much.” These nine events were summed, with higher scores indicating higher levels of job-related stress (alpha = .79).  ***Negative emotions****:* operationalized by asking police officers how often eleven statements were true such as: “I feel tired at work, even with adequate sleep,” “I am moody, irritable, or impatient over small problems,” Respondents were allowed to answer each question by indicating “never,” “sometimes,” “frequently,” and “always.” These 11 items were summed, with higher scores indicating higher levels of negative emotions. A reliability analysis indicated an alpha level of .89. | Results indicate that personally experiencing child maltreatment was associated with police officers perpetrating IPV later in life | - 9% of the police officer sample indicated that they had perpetrated physical violence toward their partner.      - Police officers who reported being victims of child maltreatment are associated with a significant 109% increase in odds of reporting perpetrating IPV toward their partners (Exp(.74) = 2.09; p ≤ .05). - Parental violence and critical incident stressors were not found to be significantly associated with IPV perpetration. - Negative emotions were found to be positively associated with IPV perpetration, whereas nonviolent values were found to be negatively associated with police IPV perpetration. - Men and white police officers displayed reduced odds of IPV violence compared to women and other racial/ethnic groups. - Marital status was found to be positive and significant. Married police officers in the sample were associated with a 121% increase in the odds of perpetrating IPV compared to nonmarital relationships (Exp(.80) = 2.23; p ≤ .05). - Interparental violence, a variable derived from social learning theory, was not found to be significant in predicting IPV perpetration in any of the logistic regression models. - Police officers who reported being victims of child maltreatment were about two times as likely to report perpetrating violence against their partners as respondents who did not report experiencing child maltreatment. The partitioned regression analyses reveal that physical abuse was associated with IPV perpetration for men but not women. - Job-related stressors were not found to be significant in either model. - In all models, individuals with higher levels of negative emotions were likely to perpetrate violence against their partners. The partitioned logistic regression models indicated that negative emotions had a greater influence on women than men. (strain theory re women) - Nonviolent values were found to be negatively associated with perpetrating IPV; officers who reported higher levels of disapproval of violence were .857 times less likely to use violence against their partners than officers with higher levels of approval. The partitioned analyses showed that women with higher levels of disapproval were less likely to use violence. | Studies that have not controlled for child maltreatment may not have provided a comprehensive test of the correlates of IPV in police families | 3 |
| **Zavala et al., 2019**  United States  (57) | Police officers | Intimate Partner violence/ spouses | Cross-sectional: analysis of already existing dataset- survey from 1997 | 1104 |  | **Domestic violence:**  Influence of job-related stress, perceived work stress, and the officers’ coping strategies | 93.2% | **Self-control** - 2 items were used to measure officers’ level of low self-control.  “I have difficulty concentrating on my job” and “feel so restless you couldn’t sit still’)  **Desire to be in control** was measured using 2 items: “I feel like I need to take control of the people in my life” and “I expect to have the final say on how things are done in my household.  **Control variables**: parental violence, experiencing child maltreatment, possessing high levels of anger, perceived work stress and exposure to critical incident stress.  (All scales seem to be conceptualised by the authors) | - Results from logistic regression models indicated that self-control was not related to IPV, but the desire-to-be-in-control was found to be positive and significant in predicting the dependent variable. - This study provided further evidence that IPV may be the result of a person’s desire to attain and maintain power and control over their partner’s behaviour. | - 7.7 % of officers were found to be perpetrating IPV (SD=0.26) - None of the models showed that self-control was significant in predicting IPV. - However, the hypothesis stating that desire to be in control would predict IPV was significant. - When self-control was included in the full model the desire-to-be-in-control remained positive and significant. |  | 3 |

## **Qualitative Studies**

| **Author, Year, Country, Reference no.** | **Responder population (e.g., fire, police, ambulance)** | **Family Relation (e.g. spouse, child, parent)** | **Topic Area** | **Method of Data Collection Type (Focus groups, semi-structured interviews)** | **Sample size** | **Analysis Method (Thematic, discourse)** | **Mental health/Wellbeing Themes/sub-themes** | **Additional Relevant Findings** | **Q. A.** |
| --- | --- | --- | --- | --- | --- | --- | --- | --- | --- |
| **Brodie & Eppler, 2012**  United States  (29) | **Police officers/Law Enforcement Officers (LEOs)** | Spouses/ partners | Explores how police and their significant others perceive the resources that have helped them be resilient with stress and other identified challenges, and how couples communicate about these issues. | Grounded theory approach: Semi-structured interviews | 7 couples | Concurrent analyses of information for themes, patterns, and categories while following analytic strategies as recommended by Creswell (2003). | - Most police officers described efforts to maintain strong boundaries between work and home, use of gallows humour, and physical exercise as coping skills. Each participant was able to describe ways their partner could offer comfort and support through assisting with household tasks, participating in co-parenting, and prioritizing alone time for the couple. Three couples utilized a philosophy accepting that life has inherent stressors. All of the couples also described utilization of social supports. All but one participant discussed a number of benefits to this career choice including camaraderie, being able to help others, pride in the job, and financial security. - All of the participants identified a formal religious affiliation, yet most described being inactive in organized activities. - Almost all participants referred to regular exercise as an integral part of stress management. - All of the police identified one or more stressors relating to their work; however, they were also able to describe several positive aspects of their work. Similarly, two-thirds of spouses could identify benefits that were specific to their spouses’ involvements in police work. These consistently centered on six main areas. Both the police officers and significant others described concrete benefits including good pay, health benefits, and (for 7 out of 8 participants) job security. Police officers also described variety in their work, especially meeting different people. Both the police and significant others referenced a sense of altruism, a sense of pride about duty, and perceived camaraderie. - Police discussed a number of ways in which they felt they were being supported by their spouses. The most frequently cited, indeed by all male LEOs, was the willingness of their wives to attend to household and children’s needs. - Each couple commented on discussions and efforts to prioritize “alone quality time” for the marital relationship, including going out together etc. - Many of the participants described discussing incidents with co-workers in a humorous way—making jokes as a way to deal with these experiences. Many LEOs and SOs talked about the sharing of funny stories or “lighter” issues in their communication about the officers’ work. - All of the participants identified social supports as a main component in management of stress and coping in their lives. Talking with friends and family members such as parents or siblings was cited most frequently. - Most couples noted that they had peer supports both within and outside of the law-enforcement community. | 6/7 couple’s communication patterns were fairly similar. Significant others expressed some frustration that LEOs provided only limited information about their daily work, despite their questions and requests for sharing. In addition, SOs recognized that LEOs were more likely to share specific details with work colleagues versus family members.  All of the couples in the study described communication as important to their own well-being, as well for the health of the relationship. The dual-LEO couple, Bill and Lisa, described a much more open stance regarding communication about work-related issues.  All of the participants identified a desire to maintain or increase communication about these issues, and each related his or her perception about actively trying to do this with their SO.  Almost all of the SOs recognized that stress was impacting the LEO when observing him or her to be more quiet than usual. It became apparent that communication levels in law-enforcement couples varied based on several factors. These included the degree to which officers perceived their spouses’ to be interested in discussing work-related issues, the content of the communication, and the officers’ motivations to communicate about specific issues. | 3 |
| **Brady et al., 2019**  United States  (43) | ICAC | Parent/children | **Mental Health:**  Does being a parent affect risk of STS in ICAC officers?  How do child maltreatment investigations affect relationships with interviewers’ own child(ren)?  How does the work of forensic interviewers affect relationships with their family and friends? | Open ended questions | 367 | Systematic content analysis | Question 1 is quantitative  **Question 2:** how do child maltreatment investigations affect relationships with interviewers’ own child(ren)?  While 23.2% (n = 78) of respondents did not have children, the majority of respondents (68.8%) who were parents reported that their work has had some effect on these relationships.  **Negative outcomes:**   - The most common response was a decreased trust of others. More specifically, 72 respondents (21.4%) used the word “protective” in their response, referring to being “overprotective” or “more protective” with their children. Interviewers with children noted that their work has made them increasingly “paranoid” or cautious to a detriment. - Many respondents with children were not only overprotective but felt as though their relationships were strained due to their work. This translated into issues of physical distance (4.5%; n = 15), such as decreased attendance and participation in school-sponsored or community events due to high caseloads and time commitments, and/or fear of running into clients/offenders, particularly in smaller, rural areas. - Emotional distance was discussed in terms of the inability to empathize with their own children’s concerns and issues. Multiple respondents (4.5%; n = 15) noted the difficulty of being empathetic to their own children after working with victims of abuse. - Among those who reported that they did not have children, three suggested that the nature of their work was the reason for this. - Other non-parents wrote that their jobs have made them more cautious of other children in their family, such as nieces and nephews, and that their careers have “provided insight” into how they will raise their own children one day.   **Positive outcomes:**   - In some cases (15.7%; n = 53), respondents reported that being overprotective had actually improved their parenting skills. Interviewers indicated that they ask more open-ended questions about their child’s day, offer more support and guidance, and use correct anatomical language to discuss private parts, along with doing so more frequently and earlier in their children’s lives. Others used their fear of their own children becoming victims as an impetus to educate them on potential dangers and to create a more open and communicative environment at home. This increased openness also led to one respondent’s daughter disclosing her own abuse. - Finally, 7.5% (n = 25) of respondents noted that their work has increased social bonds with their children, including realizing the importance of a healthy parent-child bond and encouraging them to do more activities with their children. Particularly after a difficult day at work, interviewers relayed that they looked forward to going home to tell their children how much they mean to them and “hug them a little longer.”   **Question 3:** How does the work of forensic interviewers affect relationships with their family and friends? The majority of respondents (82.3%) reported that their work has affected relationships with their family and friends, with 17.7% (n= 59) reporting no effect in this area.  **Negative outcomes:**   - Among the negative outcomes, feelings of emotional distance were most common (33.3%; n = 111), followed by miscellaneous negative comments (e.g., mood swings, marital difficulties, or psychological outcomes; 18%; n = 60), decreased trust (16.2%; n = 54), and physical distance (11.4%; n = 38). - Emotional distance was characterised as being physically present in situations, but emotionally or mentally unavailable, such as spending time with their family and/or friends but not wanting to engage them in conversation. While confidentiality was a common concern, most respondents noted that they avoided talking about their work due to the depressing subject matter, a general lack of understanding of the nature of the work, fear of traumatizing friends or family, and/or making social interactions awkward. - Physical distance was also a major concern of respondents (n = 38), mainly due to working long hours or working through weekends and lunches - time that could be spent socializing away from work. Not only were respondents physically unable to see friends and family, when they did have time off, many reported wanting to be alone or simply not having the energy to be in social situations. - Common was an in-ability to separate work from home, marital difficulties specifically as a result of their work (e.g., divorce; intimacy issues), and negative psychological outcomes, such as intrusive thoughts, and becoming more irritable and less compassionate towards the issues their loved one’s vent about, particularly in comparison to the horrific challenges their clients face. - 16% of respondents (n= 54) reported that their work has made them increasingly paranoid about the safety of their children and/or cynical and distrusting towards friends and family members.   **Positive outcomes:**   - Despite most respondents reporting some form of negative outcome, 11% (n = 36) of the sample reported only positive effects. - Increased social bonds was the most common positive theme among respondents (15.9%; n = 53). - Respondents reported that their work has encouraged closer relationships with family and friends to help overcome stress. - Being exposed to crimes against children on a regular basis has led interviewers to feel more appreciation for what they have. - Respondents also indicated a host of miscellaneous positive results of their work, including an increased sense of pride, supportive acknowledgement and praise from loved ones, and better communication skills with their children about sensitive topics. Finally, two respondents commented on how the work has made them a better caregiver. |  | 4 |
| **Bochantin et al., 2016.**  United States  (44) | Police officers and firefighters | Dependents and relational partner: spouse, child or both. | Public safety employees’ (PSEs) (police officers and fire fighters) experiences with work and family as well as the experiences of their family members. | One-on-one semi structured interviews (x36) for the police officers and firefighters; and a mixture of family interviews and focus groups (8x 5-7 members at a time). | 95 Participants in total:  36 interviews: 18 police officers, 18 firefighters  Traditional families with spouse and at least 1 child = 18; single-parent families because of divorce = 7; single families because of being widowed = 2; married partners with no kids = 8; female participant who was married and pregnant during the study = 1.  Homosexual = 9; heterosexual = 27;  Men = 26; Women = 10.  All but 3 were from urban, suburban areas.  Patrol officers = 9; school resource officers = 2; sergeants = 3; detectives =3; chief of police = 1.  Standard firefighters = 13; lieutenants = 3; battalion chief =1; chief = 1.  59 family members: 27 spouses (19 wives/partners, 8 husbands/partners, 2 ex-spouses); 32 children, 20 of which were under 18; 9 of which under 13 (equal number of male and female children) | Grounded metaphorical analysis | - **Competition:** More than any other type of metaphor, participants compared their experience in dealing with work and home as being some sort of a game, war, or challenge. Some participants were so frustrated with the competition between the two domains and some so overwhelmed with the possibility of danger. - **Nature/preservation:** When describing the relationship between work and family, many participants used nature or preservation metaphors to help portray their feelings. There was a total of 21 different nature/preservation metaphors that participants made reference to in order to describe the relationship between work and family, including, “a blizzard,” “an island,” “a tree,” “a shadow,” “thunderstorm,” and “a spider web.” Because nature is full of examples of successful and unsuccessful growth and preservation, and full of examples of disaster and beauty, many use nature as a source of metaphor when trying to understand life. Additionally, the nature metaphors that were uncovered include both positively charged and negatively charged metaphors. - **Ambiguity:** Whereas many participants used metaphors of competition and nature/preservation to describe the relationship between work and family, several others used metaphors that reflected ambiguity with regard to how they feel about and make sense of the relationship between work and home. Metaphors such as “wild card,” “mirage,” “darker side of the colour wheel,” and “morning fog,” were used by participants. Renee, the wife of a police officer employed the metaphor “darker side of the colour wheel” to express how she makes sense out of how her husband handles the relationship between work and family. She talked at great lengths about scheduling and how it could either be good or bad for family/work life.   **Conclusions:**   - Current understanding of existing work–family constructs (i.e., segmentation, spillover, conflict, etc.) are likely understood and experienced differently for members of the public safety profession and we seem to be moving away from the dominant construct of “balance;” - Emotional labour is being performed by both PSEs and their family members. - **Work-Family constructs:** Segmentation is the intentional separation of work and family roles such that the thoughts, feelings, and behaviours of one role are actively suppressed from affecting the individual’s performance in the other role. This paper has found this to be a valuable strategy whilst previous research sees it as destructive and negative for example children withhold their “true” feelings from their PS parent when they would put on “brave faces” so that their mom or dad would not go to work stressed about how they were feeling about the job. In many cases, these children were wracked with fear over the nature of their parents’ job. Thus segmentation, as revealed through nature/ preservation metaphors, can be quite productive for PS families. - Metaphors reminiscent of the past construct of spillover were also observed through the category of competition metaphors, e.g., they would describe situations where their parent or spouse would have a difficult time “turning off” their work personality while at home and would in many cases treat children unfairly or severely. It would seem that many PSE’s experiences a form of role conflict where they have a difficult time transitioning from one sphere (i.e., work) to the other (i.e., home) and instead, blend the two which often results in anti-social behaviours at home. - Very few references to balance metaphors, suggesting that it’s not necessarily desirable or possible to attain for PS families and may be many other types of professions. - **Emotional Labour:** Children spoke about putting on “brave faces” or pretending to be emotionally stable for their parents to ensure they did not go to work stressed. Many of the children commented that while they were “afraid” for their parents at work, they would never admit this to their parent, thus engaging in surface acting. - Police/firefighter parents also engaged in emotional labour when children or spouses would ask them to share stories from their workday, which would encourage the negative experience of spillover. Instead of telling the truth about any of the atrocities they may have seen at work, they would instead share stories using humour and benign examples. Certain situations require their emotions to remain bottled up. - Moreover, family members engage in emotion work by denying their feelings to their PSE loved one. Because PSEs do not have the ability to talk about their job/emotions at home, they can become emotionally detached. This could potentially explain the higher divorce and alcoholism rates among PSEs, particularly among police officers. |  | 5 |
| **Helfers et al. 2020**  United States  (65) | Police | Children  Ages 13-17 | Lived experiences of being a child of a police officer | Semi-structured interviews | 19 | Qualitative phenomenological approach | - **Positive protective parenting** – children commented on feeling a heightened level of a protection from their ER parent and this was considered a positive thing. - **Negative protective parenting** restricting freedoms of children – children found ER parent to be overprotective intrusive and limit activities they could do or involvement in social media. - **Great resource** – ER parent was considered to be a good resource of information for the child and helped them become more knowledgeable about their rights and legal processes. - **Worry** – children reported concern worry for their parent because of their occupation. Subthemes under ‘Worry’- **Parent getting hurt** – children worried their ER parent would get hurt on the job- **Children being harassed** – children reported being bullied or harassed because of their ER parent’s job. **Unfair treatment** – children worried about their police parent being unfairly treated in the media and unfair comments being made about their parent (police are violent, racist etc)-**Parents not over supportive of policing as a profession** | Children were torn between appreciating their parents because the police job helps the children be safer than others, and the feeling of losing out on something in their childhood due to a loss of freedom.  Many emotional and psychological impacts of worry children display, and harassment experienced of feeling of parents’ police job not being understood by the public. | 6 |
| **Karaffa et al., 2015.**  United States  (30) | Police officers | Spouses | **Impact of police work on spouses:**  Determining the types of difficulties evident in police marriages. | **Open-ended items:** Re: the most difficult aspect of being a police officer or the spouse of a police officer. | 82 officers and 89 spouses | **Not reported** | - Spouses reported feeling pride about being married to an officer, they also noted financial concerns, Work-Family-Conflict (WFC), and law enforcement-specific stressors, such as negative public attitudes toward police. Officers and spouses reported relying on friends and family for support more than on professional sources. - **WFC:** One respondent from the spouse group indicated that the most difficult aspect of being married to a police officer is ‘‘*dealing with scheduling conflicts*.’’ She stated, ‘‘*It seems that our families are always trying to accommodate his schedule. I’m often embarrassed about it and feel guilty that others have to be flexible for it, but we can’t be flexible for them*.’’ - Another spouse explained, ‘‘*Because our work hours are so different, we don’t get to see each other very often. That makes it hard since we have only been married for two months.*’’ Issues with work hours may be more pronounced when couples have children: - For example, another respondent in the spouse group said, ‘‘*Sometimes I feel like a single parent trying to get the children to all the events they are involved in*.’’ Several officers also emphasized the stress associated with WFC. - Some spouses also indicated that stress related to scheduling and WFC may depend on the couple’s financial situation or the officer’s position. - **Communication/ emotions:** One respondent said: *I feel that in order to maintain his own sanity and emotional well-being, my spouse detaches himself from his feelings. This causes difficulty between us because he has become essentially unable to get in touch with any emotion at all.* Another spouse explained that the most difficult aspect of being married to a police officer is: *His inability to communicate and share emotions with me. It’s not difficult to be married to a police officer.* - An officer stated that his job had ‘‘*robbed him of [his] compassion and the ability to feel emotion*,’’ and another agreed that ‘‘*cops tend to suppress their emotions—they are expected to.*’’ - Some respondents indicated that officers’ tendency to keep their experiences and feelings private may be a way of protecting the family. - **Peer socialisation:** A spouse explained*: I have lost all trust in him from putting his co-workers before his family. He developed bad habits after working with a certain group of officers and other employees over several years. Basically, he was living a double life and we have come very close to divorcing. I’m the first to understand the closeness of bonds they need to be safe and successful. But many officers do not know where to draw the line and incorporate their family into the life of an officer. After all, it is also the ‘‘life’’ of their children and wives as well, not just the officer”.* - **Resources used:** One spouse reported: *Police families do not know a lot of each other. We’ve been here for a few years, and I only know about families. The police department and their families are not brought together enough so you can meet the fellow officers and families.”* - **Personality:** Several respondents from the spouse group mentioned that personality changes or emotional displacement were the worst aspects of being married to an officer. One respondent explained, ‘‘*The job is never over; it’s a part of who he is as a person. Being an officer has changed my spouse. He is not as joyful and carefree as he once was in college. He trusts no one*.’’ Spouses indicated that their significant other ‘‘*tends to stay in ‘police mode’ at home*,’’ and another described ‘‘*the callousness that [her husband] has gained towards many emotional situations over the years as a police officer.*’’ - **Public Attitudes:** A spouse indicated the difficulty of *‘‘seeing the erosion of respect for police officers that has occurred over time—knowing that the uniform and the squad make him a target to some.’*’ She admitted that ‘‘*his squad has been egged, paint-balled, and had glass broken just sitting parked in front of our house.*’’ Being married to a police officer may also affect spouses in their own peer relationships. A respondent from the spouse group explained the difficulty of *‘‘having to live by a higher standard than family/friends live by, which may cause conflicts within these relationships*.’’ Some officers also recognized the benefits of maintaining friends outside the department. One reported: *The most difficult thing for police work is learning to have outside friends. Most stress on officers comes from hanging out with other officers all the time.* - **Departmental Issues:** Several spouses and officers cited that issues related to departmental politics were the worst aspect of being an officer or a police spouse. A spouse respondent explained that officers are ‘‘*not encouraged to be a family-man/woman,’’* and another concluded that officers face a ‘‘*lack of respect, support, and understanding from upper levels of administration.’’* Similarly, an officer reported that the ‘‘*lack of concern by supervisors about family problems’’* - **Spirituality:** Many police families rely on spiritual beliefs to manage the stressors associated with being an officer or a police spouse. An officer reported ‘‘*I enjoy my career as a police officer, and believe that my faith has helped me deal with a lot, without having to put a lot of stress on my family.’’* |  | 3 |
| **Landers et al., 2020.**  United States  (32) | Police | Spouse/partner | Mental Health: Exploring the lived experiences of secondary trauma among partners of law enforcement professionals. | Interviews: phenomenological | 8 | Transcendental phenomenological inquiry | - **Theme 1: Types of Traumatic Event:** Participants identified numerous types of traumatic events to which their Law Enforcement Partner spouses had been exposed, including death scenes involving citizens, infants, or fellow officers; injury of fellow officers; emergency response situations, such as disasters and fires; domestic disputes; medical emergencies (e.g., performing CPR); and motor vehicle accidents. - Although all of the incidents were traumatic, some participants noted that the LEPs were affected differentially by different types of traumas; events involving children, suicide, and officer death were reported to have the greatest impact on the LEP from the perspective of the spouse. Most partners talked about the LEP witnessing or responding to violence or death. Others described direct injuries to the LEP or a colleague, such as the death of a partner, a broken shoulder while pursuing or apprehending suspects, and being severely injured to the point that survival was in question for a few days. Although each exposure was considered traumatic according to the partner of the LEP, not all were equal in their impact. - Emotional and physical reactions to LEP trauma are experienced by LE spouses as well. Participants in this study described providing a supportive role by offering emotional support, caregiving, being flexible, and communicating effectively. Couple coping strategies are employed after exposure to a traumatic event, including increased communication, mutual support and listening, engaging in activities together, and renegotiating roles and responsibilities. - Partners of LEPs and the couple’s relationship were affected by secondary traumatic stress. Secondary traumatic stress was manifested in both emotional and behavioural ways, as evidenced by how partners described the LEP’s emotions and behaviour (and their own reactions to the trauma). LE couples displayed a range of coping behaviours in response to the trauma, while partners operate within a supportive role. - **Theme 2: The ripple effect of trauma:** Participants described a wide range of emotional reactions in the LEP following the traumatic event(s), including mood changes, detachment, internalizing, hypervigilance, reexperiencing or replaying the event, anxiety, and avoidance. Many participants talked about the emotional impact of trauma on the LEP. Other participants described the LEP’s behavioural reactions to trauma I.e., hypervigilant behaviour that signified they were always on guard and cognizant that the next traumatic event could occur at any time. - Participants described their own emotional reactions to secondary trauma. More generally, participants described experiences with nausea, intrusive thoughts, anxiety, shaking, confusion, mood changes, fear, and worry stemming from their own responses to the LEP’s exposure to traumatic events. Participants described experiencing a sense of “worry” for both themselves and their partners. - **Theme 3: The Strength of Couples and How They Cope with Trauma:** *Individual coping:* Participants described coping mechanisms that entailed both seeking and providing personal support within the LE community, whether in spousal support groups or from other LEPs and spouses. - *Couple coping in response to trauma.* Participants reported coping as a couple by communicating about exposure to traumatic events, providing mutual support and understanding to one another, being conscious about coping as a couple unit, demonstrating flexibility, and prioritizing quality couple time. Increasing communication, shared exercise, and maintaining a simplified or routine schedule were other modes of couple coping. - *Supportive role in response to trauma.* Participants described playing a supportive role to the LEP by being empathetic, taking on additional responsibilities, providing emotional support, caregiving, using humour, providing encouragement, being flexible, and adapting to the needs of the LEP. Openly communicating about traumatic event exposure and processing trauma were important for many of these LE participants. They understood that processing traumatic experiences took time and that there were no shortcuts. |  | 6 |
| **Menendez et al., 2006.**  United States  (45) | Firefighters who participated in rescue efforts following 9/11. | Spouses of NYC firefighters | **Mental Health:** This study attempts to identify patterns in survivors’ responses to the events as well as any coping mechanisms that the participants used to manage their exposure to the tragedy. | **4 focus groups with 5-6 spouses per group (2 hours per session).**  Demographic forms with a brief questionnaire. | 21 spouses  All female  Ages ranged from 31-49, married from 8-23 years.  1 participant was separated following 9/11.  All Caucasian, 18 catholic, 2 Jewish, 1 declared no religious affiliation.  All had children, ranging in age from 3 months to 17 years at the time of the interview.  17 of the women worked. | Analysis was done on an ongoing basis to uncover themes, ideas, and concepts with notes made in margins of the interviews when a concept was identified. | **Firefighters:**   - “It was a war; we were on the home front holding it down.” The initial days of the event were the most difficult, as expressed by uncertainty for the safety of spouses at Ground Zero and painful moments of separation when husbands left precipitously for rescue. Many of these wives reported not hearing from their husbands for days, and one wife stated that her husband did not return home from rescue efforts for 2 weeks following the event. His communication was frequent, however, regarding the devastation surrounding him*. All participants reported that their husbands knew firefighters who had perished.* - Common theme was the husband’s comment about the odour at the rescue site. One woman stated, “He couldn’t get beyond that smell . . . that’s all he talked about.” Another woman was told that “there were body parts all over . . . you can’t possibly imagine.” In general, however, many of the women expressed concern that their husbands did not talk about what had happened during their tour of duty at Ground Zero. - Some reported that they saw their husbands crying for the first time, but many husbands did not talk and most refused counselling. One woman expressed outrage that counselling was not mandatory. - Some wives demanded that their husbands and families receive counselling. One woman stated that counselling “helped him so much. He saw things as only black or white, there was no grey. He just couldn’t justify why he was living and that others had died.”   **The Children:**   - One participant reported that her son, age 11, would become very frightened and worried if her husband returned home late. Other mothers reported that children needed to be reassured as to where their fathers were and what time they would be home and required reassuring phone calls that they were okay. - A child of 7 became very distraught when her father informed her, he was going back to work after the Christmas and New Year holiday following 9/11. The mother described it as “a meltdown.” - Mothers of older children did report speaking with them about the events, and mothers of younger children tended to be more cautious. Many respondents reported that their children attended funeral services for deceased firefighters. Initially, most women viewed this as an important way to show respect and honour the fallen firefighters. Many became overwhelmed by the sheer number of funerals and worried about the adverse impact on their families.   **The partners:**   - Many reported insomnia and anxiety early on after 9/11. 80% of the women reported no change in their overall health. Three of the women reported good health, but feeling more “*emotional, stressed, or nervous*.” Another woman with numerous medical problems prior to 9/11 described them as more pronounced after 9/11. 5/21 women, none of whom had previous counselling, sought treatment post- 9/11. - Most of them did report speaking to other firefighter spouses to gain information and to seek support, though initially they did express worry and trepidation about speaking to women whose husbands were reported missing or who had died. This was described by some as “*survivor’s guilt*.” In terms of spirituality, although many of the respondents reported no change, one woman stated that “*right after 9/11 I looked forward to church, I found the quiet time comforting.”* - Two others reported increased spiritual practices, but not from a formal perspective. *“I pray more but don’t necessarily go to church*.” Another woman stated that she had become more spiritual through the practice of yoga. - Interestingly, however,4 women reported that they had decreased their religious practices. One woman stated, “*I have lost my faith; I question my belief to the point where it drives me crazy. This has been the greatest change for me.”* - In terms of how life has changed for them, many women described their marital relationship in the initial week’s following 9/11 as strained. One woman stated that her husband had an affair post- 9/11 and that they were in the process of divorcing. - 24% of respondents at interview reported still feeling a strain in their marital relationships. Most of the participants, however, reported no significant changes except that they not only appreciated people more, but enjoyed being around them. They tried to listen better, and they said that they were more open to people and situations. - Many did report worrying for their children’s future. Less than 10% described thinking negatively about things they would take for granted in the past, like traveling on planes or even going into Manhattan. All the women reported being conscious about not imposing their fears on their children.   **Conclusions:**   - Most of the wives received their psychological support from other women, particularly other firefighter spouses. They helped each other deal with their anxiety, fears, and sadness. They kept communication going about their husband’s whereabouts concerning rescue efforts, and they spoke to each other about their husband’s psychological well-being, well after rescue efforts had ended (most did not receive counselling post 9/11. - All the women reported a need to “hold the fort down” and to retain a sense of normalcy in a very abnormal situation – they spoke about the importance of focusing on family and home life. - The stress of trying to maintain the routines of day to day living certainly placed a heavy burden. - Increased anxiety was common around the anniversary of 9/11. - Many worried about the physical health of their husbands, with safety being an issue, and had specific concern about long-term respiratory problems. They worried about their spouse’s emotional health, with many reporting mood changes and/or excessive drinking off duty. - Respondents were all keenly aware of the importance of being available to give support to their children. This has been identified as an important factor in distinguishing traumatized youngsters with good outcomes from those who did not fare as well. |  | 4 |
| **Porter & Henricksen, 2016.**  United States  (31) | First responders (law enforcement, fire service, emergency medical service) | Spouses | **Lived Experiences**: Identifying significant barriers and stressors that exist within the first responder family system | **Phenomenological approach: semi-structured interview.** | 6 Spouses: age range from 27 to 41 years old. 5 female spouses and 1 male.  3/6 police, 2 fire department and 1 = emergency medical services. 5 = white; 1 = Hispanic.  5/6 employed outside of the home ; 1/6 stay at home parent. | Horizontalization/ phenomenological analysis. (quasi statistics) | **5 MAIN THEMES:**   - **Safety:** 6/6 participants endorsed safety as a key component in the first responder lifestyle. The participants were able to recall some humorous examples of how safety plays a major role in their lives, but there are also some sobering scenarios in which the issue of safety is paramount. Safety from physical injury is a major theme that emerged from the data. The participants, while light-hearted at times, were always fearful of the ‘‘worst-case scenario’’. 4/6 participants cried while discussing their safety concerns and 2/6 were very solemn in describing fears they have regarding their spouse’s occupation. - **Stress:** All six participants described the stress that is associated with the first responder career. They indicated that the amount of stress upon both the first responder individual and family can lead to difficulties at home, and in several cases, the spouses stated they felt directly responsible for helping relieve stress and encouraging their first responder spouse. The participants also indicated that constant communication was crucial to maintaining their marriage. - **Pride:** A major theme that was endorsed by six of six respondents. Each participant relayed stories and examples of how their spouse had positively impacted the community and the pride they felt as first responder spouses. Jane stated, ‘‘It can be stressful but it’s also something to be proud of.’’ Each of the participants had many examples of how their spouse’s occupation instils pride within the family unit and individually as a first responder spouse. - **Civic mindedness:** A subtheme of pride referred to as civicmindedness emerged as the participants recalled their experiences as first responder spouses. In addition to the pride they feel as a spouse, they discussed a sense of duty and responsibility to the community. - **Identity:** All the participants expressed a sense of pride in the occupations of their spouses; however, this pride extends further into the identity of the first responder family. Five of six participants endorsed this theme and as Elise described, ‘‘I don’t just feel like it’s him out there, it’s me too. We are a team, the whole department is a family, and you can’t help but feel a part of it.’ - **Finances:** The participants all discussed financial hardships that they experienced in their household. | Participants of this study described an essence, or way of being, through their lived experiences  **Main findings:** One of the major findings of the study was how deeply the participants identified with their spouse’s career.  A central message for marriage/couple and family counsellors, which was conveyed through the interviews, is that in order to best serve this population, we must not judge or be overly critical. | 5 |
| **Regehr et al., 2005 (a)**  Canada  (26) | Paramedics | Spouse | Impact of trauma exposure on spouses of paramedics | Semi-structured interviews | 14 | Thematic | - Aftermath of traumatic events and work stress for paramedics has a ripple effect on families. They often subjugated their own needs for their paramedic partner and there is little support offered for spouses (formal or informal). - **Everyday Hassles:** Traumatic events occurring within the context of a stressful work environment that disrupted and spilled over into family life. Specifically, pressures of shift work – unpredictable nature of work meaning family time is compromised and family responsibilities are not evenly distributed. Spouses felt like single parents and often elected to work part time to endure consistent childcare. Everyday pressure of job impacted on time couples got to spend together, sex life and social activities. - **Concerns about dangers of the job:** Spouses worried about dangers of the job such a violence towards paramedics who were not armed like police, driving conditions in bad weather, exposure to disease (HIV, SARS) - **Effects of Stress and Trauma on the Paramedic:** They reported their partner experiencing mental health symptoms such as depression, PTSD (nightmares), anger and physical somatic conditions and impact on sleep. They reported their partner’s personality changing (anger, less caring, more withdrawn, hardened) - **Impact on Family of Stress and Trauma:** Family attempts to manage the mood states of their partners in aftermath of trauma. Tried to avoid triggering problems, creation of fragile atmosphere in the house. Described vicarious trauma of listening to paramedic’s description of trauma experienced. Alternatively, paramedics would not share issues and became closed off. - **Coping Strategies in Spouses of Paramedics:** Spouses and children tried to join paramedic on ride alongs or medical courses to understand role better. Avoidance strategies to sidestep emotional issues – caused some spouses to subjugate their own emotional needs. Spouses tried to develop independent interests and friends. - **Supports available:** Reported peer support and some professional support for the paramedic. No support offered or available to spouses. |  | 5 |
| **Regehr 2005 (b)**  Canada  (27) | Firefighters | Spouse | Effects of emergency service work on the spouses of firefighters | Semi-structured interviews | 14 | Thematic (Strauss and Corbin, 1990) | - Spouses highly supportive and proud of spouses work in firefighting, however areas of challenge focused on effects of shift work on family life, transfer of firefighters reactions to dangerous and traumatic events on the family, lack of social support experienced by spouses because of rationalisation of fire service and less community integration with spouses more isolated. Spouses worried about their partners at work and risk of situations they were exposed to. - **Pride and glory: firefighting as a profession**   **-** Pride in firefighting profession  - Positive personality traits of a firefighter  - Reflected glory  - Own stresses and needs are secondary   - **‘‘Mr. Mom’’ and the single parent: shift work and family**   **-** Flexibility regarding time and care of younger children  - Discipline issues for older children  - Missed family events  - Lack of couple time   - **‘‘Guys hang out, not women’’: social supports**   **-** Strong network for firefighters  - Isolation for wives   - **‘‘You try to read them’’: responding to stress and trauma**   - Managing fear  - Managing stress and trauma spillover  - Support for firefighters, not families | Nature of firefighting exposes families to forms of ambiguous loss: 1) physical absences created by shift work, 2) emotional and physical absences created by firefighter camaraderie at exclusion of families, 3) emotional absences stemming from trauma responses, 4) loss of certain/secure future when faced with death or injury of other firefighters.  These challenges no longer ameliorated by social supports because of larger more complex organisations – challenge to develop and implement programmes that encourage mutual aid and social support among families. | 4 |
| **Richardson et al., 2016**  United States  (69) | Firefighters | Spouse – specifically 9/11 widows | Social support and Post traumatic growth | Online survey – open text box asking how widows met other widows and how/if the peer groups had helped with personal healing related to 9/11 | 55 | Phase one -Interactive synthesis (3 coders) (Huberman and Miles 1994)  Binary coding system to categorize qualitative data into quantitative data themes. | Themes of informal support and how it had helped widows.  Coding of qualitative responses revealed 11 themes.  Shared pain and grief  The most frequently cited theme was that of shared pain (56%), followed by friendship (26%), child rearing and support (23%), and emotional strength (21%)   - **Shared pain and grief** - Sharing the same trauma experience and therefore knowing and understanding exactly what each other is going through. - **Friendship -** Camaraderie and bonding with each other; development of lasting friendships. - **Child rearing and support** - Ability to discuss issues regarding their children and support each other as single parents. - **Emotional strength** - Ability to draw strength from each other to deal with emotions and feelings. - **Trust** - Ability to discuss all manner of topics openly and honestly, without judgment. - **Paperwork -** Assisted each other with the myriad of paperwork and forms that needed addressing. - **Vacation** – Taking vacations together - **Moved on** – No longer gathering with other widows. - **Feel less alone** - Feeling less isolated. - **Learn from each other** – provide a resource of information. - **Talk about loved one** – Ability to discuss deceased husband. |  | 4 |
| **Roth et al., 2009**  United States  (28) | Emergency Medical Service (Paramedics and EMT) | Spouse (11 interviews) and parent (1 interview) | Impact of Emergency Services work on family system | Semi-structured interviews | 12 | Phenomenological approach – 2 independent coders to create categories, these categories used to code all interviews. Checked for consistency by two other researchers | - Stress associated with a career in EMS can impact the work–family fit and function of the family system. The authors found that families in this study coped with challenges associated with their family member’s EMS work through negotiating role responsibilities, maintaining open communication, developing their own interests or hobbies, giving their EMS provider “space” upon return to home life, and providing support by listening and helping the EMS provider process his or her reactions to difficult work. In addition, family members reported concern over their EMS provider’s safety. - Overall, the findings of this study were encouraging in that despite the challenges to work–family fit within the family system, families of EMS providers appear to be resilient and adaptable   **Impact of Shift Work on the Family System**   - **Shift work, holidays and Social Life –** interruption of shift work to holidays and family’s social life. Unpredictability of overtime and not knowing when spouse will be home. Shift work on weekends meaning children sport/social activities are missed by paramedic. - **Changes in marital and parental roles** – spouse had to take on more housework and duties compared to EMS partner and took on more parental roles. Shift work affected time for couples to have intimacy. - **Rhythms of homes life** – rotating shifts meant family life had no set scheduling and family had to fit around EMS partner sleep pattern. One spouse saw this as a positive meaning life was not dull or monotonous.   **Coping with the Impact of EMS work on the family system**   - **Providing emotional support and space** – spouse provided emotional support, often subjugated their needs to their EMT partner, ‘staying out their way’ until they were ready to speak. - **Cognitive strategies** – ‘going with the flow’ accepting the ups and downs as part of life. Spouse prepared themselves for worst outcomes that they would most likely go to social events alone or disappointment. - **seeking social support** – spouses sought support from family and friends. EMT schedule gave spouse freedom to see friends. Balance of friends inside and outside EMT environment. - **Negotiating family role responsibilities** – some couples did this more successfully than others, taking turns in childcare and managing schedule to give time for family. Some spouses reported they felt most household and parenting duties fell to them. - **Developing your own interests** – spouses developed their own interests in the time the EMT partner was away which helped to balance their lives - **Concerns about Physical safety and job risks** – discussed concerns about health risks such as physical safety, contagious disease risk, needles etc and poor weather conditions when driving. | EMS providers and families can be educated about family functioning and the dimensions that contribute to work–family fit, including communication strategies, problem-solving techniques, and ways to balance family roles | 4 |
| **Waddell 2020**  Australia  (51) | Fire, paramedic, police | Partner | Lived experiences partners of veterans and first responder with PTSD | interviews | Ten participants were partners of veterans, five of paramedics, five of fire-fighters and two of police officers | Qualitative phenomenological approach, inductive thematic analysis | - **Protecting the family unit** – participant wanted to ensure the wellbeing of all family members. Impact of PTSD on relationship -challenges in managing and coping with the person's social and emotional withdrawal, breakdown in communication and unpredictable mood swings with regards to PTSD. Loss of intimacy and sense of grief for loss of former relationship. Participants described the unpredictable nature of triggers and the need to monitor their own behavioural responses they retained the peace by avoiding conflict, although at the expense of suppressing their own need. Partner took on new family roles in decision making, earning, family organisation. - **Need for support** – partners wanted to find the right support for their FR partner with PTSD. Reported sense of loneliness and misunderstanding from others of PTSD, felt judged by others for remaining in the relationship. Highlighted that peer support was not easy to come by and had to find groups of people with similar experiences and learn from them in coping with a partner with PTSD. - **Barriers to support** - descriptions of lack of services and lack of concern for the participants and children; most intensely felt from the government, healthcare providers and the veteran or Emergency Services First Responder organisations. Lack of recognition of support function by providers that partners played, and lack of support offered by organisations. - By protecting the family unit, the person with PTSD was both supported and encouraged in their recovery. Despite the strength of commitment, the participants conveyed to maintaining the well-being of their family, they needed emotional, social and organizational support to do this. They described considerable cultural and organisational barriers, to accessing much sought-after support. | For partners of ESFRs, their capacity to manage triggers was complicated by their partner's continued exposure to workplace stresses  Note sample does include veterans, but quotes come from all first responders also and piece highlights first responder issues. | 6 |
| **Watkins 2021**  United States  (33) | Firefighters | spouse | Family dynamics, firefighter shift work and sleep | Focus groups | N=48  10 focus groups  38 firefighters in 7 focus groups  4 fire chiefs in one focus group  6 family members in two focus groups | Grounded Theory | - **Work shift schedule and accumulated sleep loss impacting social and family relationships** – accumulated sleep loss over the years and the toll it has had on their personal relationships during time off   subthemes  (a) Increased accumulated sleep loss due to an increased volume of non‐ emergent calls  (b) Emotional unavailability and a lack of communication during time off  (c) Compounded responsibilities for spouse – increase in responsibilities at home on spouse and tensions created in relationship  d. Inflexibility of family power dynamics – issues of roles and authority, spouse wish to remain in charge when firefighter back from shift.   - **Prioritization of home and family obligations over reducing sleep debt** - Married firefighters, including married battalion chiefs, noted a compulsion to stay awake after a shift so that they could not only care for children or the household, but also attend social activities such as watching their child play a sport or attempt to watch a movie with a spouse. - **importance of sleep support at home for firefighters -**   Spouses discussed creating opportunities for their firefighter to sleep – changing bedroom lighting, driving to events so firefighter could rest – note lack of firefighter presence in their sleep time as a burden but also note the desire not to have a tired or moody partner from lack of sleep. | Consequences of poor sleep and compassion fatigue add to family conflict at home.  Participant responses highlight the need for increased efforts to reduce firefighters' call volume, as well as additional training in sleep hygiene practices that maximize recovery to improve the mental and physical health of firefighters while also preserving time during days off from work to promote family and relationship cohesion  Authors argue findings highlight the need for educational opportunities for spouses to support their mental health and preserve communication with their partner.  Note that single and divorced firefighters found it easier to sleep at home, and particular pressures on those that were married. | 6 |
| **Wheater et al., 2017**  South Africa  (34) | Emergency Services | Spouses | Secondary trauma of emergency services spouses | Semi-structured interviews | 8 | Thematic (Creswell 2009) | - **Impact of emergency service work on marital relationship** – long working hours, inflexible shifts, high stress, communication challenges – impact on marital relationships. These pressures reduced the quality of marital interactions and created negative feelings about the relationship. Decreased quality time because of working hours. Unpredictability of calls and shifts impact on relationship and family responsibilities not equally shared. However, some spouses were proud of their partner and respected the work that they did. - **Impact of secondary trauma on the marital relationship** – secondary trauma from the spouses need to make sense of and emotionally connect with their spouse. Often disturbed or upset hearing about the emergency service personnel’s experienced, especially when involving children. Do experience symptoms of secondary trauma such as being helpless, sad, confused, or worried. |  | 5 |
