# Supplementary material for: The mental health and wellbeing of spouses, partners and children of emergency responders: A systematic review
Source: PLoS One. 2022 Jun 15;17(6):e0269659. doi: 10.1371/journal.pone.0269659 (PMC9200352; doi:10.1371/journal.pone.0269659)
Supplement: S1 Protocol — (PDF) [file pone.0269659.s003.pdf]

Citation

Noa Solomon, Marie-Louise Sharp, Nicola Fear. What evidence do we have about the mental health and well-being of the families of first responders and those in public safety roles?. PROSPERO 2019 CRD42019134974 Available from:
https://www.crd.york.ac.uk/prospero/display_record.php?ID=CRD42019134974

Review question

What evidence do we have about the mental health and well-being of the families of First Responders and those in public safety roles?

Searches

Relevant studies published in peer-reviewed journals will be identified through electronic searches on MEDLINE, PsycINFO, EMBASE Web of Science, PILOTS and EBSCO CINAHL databases.

Where appropriate the reference lists of included studies will be used to identify possible additional studies. Relevant academic peers will be asked to view the reference lists and indicate any other possible missing studies.

Grey literature will be searched through a Google Web search, Google Scholar and reports identified through stakeholder engagement.

Types of study to be included

o All studies published in peer-reviewed journals are eligible for inclusion into the review

o All studies/reports deemed as ‘grey literature’.

o Empirical studies

o Research using quantitative methodologies

o Research using qualitative methodologies

o Research using mixed methodologies

Condition or domain being studied

Mental Health and Wellbeing.

Participants/population

Families of First Reponders or those in public safety roles.

Intervention(s), exposure(s)

Reviewing all quantitative and qualitative data: observational, interviews mainly.

Comparator(s)/control

Families of people in other roles.

Main outcome(s)

Mental health outcomes include:

o Common mental health disorders (depression and anxiety disorders)

o Post-traumatic stress disorder (PTSD)

o Alcohol problems (hazardous drinking, misuse, abuse, dependence etc.) or substance misuse

o Stress (Traumatic Stress reaction, burnout, secondary traumatic stress, compassion fatigue etc.)

o Well-being outcomes are defined to encompass both hedonic and eudemonic traditions including concepts of emotional well-being and personal development. Hence encompassing studies that assess factors including:

o Positive relations with others, autonomy, purpose in life, personal growth (Ryff 1989)

o Social support and relationships (Larson 1993),

o Satisfaction with life, long-term good emotions and an absence of unpleasant emotions (Diener 2000).

o Resilience

o Employment status

o Short/long term sick leave

o Financial outcomes such as debt

Measures of effect

Not applicable.

Additional outcome(s)

None.

Measures of effect

Not applicable.

Data extraction (selection and coding)

o Titles and abstracts from electronic database and grey literature searches will be screened for possible relevance by a single author. Where there is uncertainty by the single author, the title and abstract will be sent to a second author to be screened, and eligibility will be decided upon - all this will be done via the Covidence software which allows up to three researchers to vote on each paper.

o 10% of studies will be assessed by a second reviewer at the screening stage to assure the eligibility process.

o One author will undertake reference checking. Full papers will be obtained that appear relevant. Another author will independently check the full papers against the inclusion criteria with discrepancies resolved by discussion and arbitration with project team.There will be 2 researchers in total.

The data we will extract will be age, gender, profession, how long they have been in their current role, relationship of family member, mental health and well-being impact on the family member.

To Exclude:

Papers examining mental health, well-being outcomes in families of general emergency service roles with no first responder/public safety responsibilities
Papers assessing physical health of families of FRC/ those in public safety roles

Papers assessing families of Armed Forces First Responders

Risk of bias (quality) assessment

A quality appraisal tool was adapted to critically evaluate the articles by drawing on relevant questions regarding recruitment, method and design, from the Consolidated Criteria for Reporting Qualitative Research (COREQ) (Tong, Sainsbury & Craig, 2007) and 'The Quality Assessment Tool for Observational Cohort and Cross-Sectional Studies' (NIH Quality Assessment Tools). Six questions, comparable across qualitative and quantitative studies, will be applied to each article. A score of 1 will be given for every 'yes' response and a score of '0' for every 'no' response. The studies will be rated as 'good', 'fair' or 'poor' quality depending on their score (scores of 5-6, 3-4, 0-2 respectively). The quality assessment will be completed independently by two of the authors, with discussion of any discrepancies until an agreed consensus score is reached.

Strategy for data synthesis

Two researchers will accumulate the quantitative and qualitative papers and separate them into different tables with the following variables:

o Authors

o Title

o Overview of study characteristics

o Study type (Prospective, Cross-sectional, interviews etc)

o Size and response rates

o Branch or branches of First Responder/public safety family population

o Theme measured (Mental health, well-being)

o Specific outcome measures (e.g. Depression)

o Types of measures used (e.g. PCL-C etc)

o Results

Quantitative data will then be synthesised using narrative methods and tabulation – prevalence of mental health statistics and well-being measures will be classified as non-significant, small, medium large (guidelines on classifying size effects, Cohen 1992) and direction noted.

Qualitative data will be synthesised using narrative methods, themes and tabulation/diagrams where appropriate.

Any sub-group analysis will be reported. (Age, gender, role, relationship to First Responder etc.)

Limitations of any of the studies will be addressed as well as ways of improving in the future.

A Quality Assessment will be conducted at the end asking the following questions:

Is the question clearly stated? Are the methods easily reproducible? Was a comprehensive search conducted? Was any bias (e.g. publication or selection bias) avoided? Was there duplicate study selection and data extraction (meaning was there a comprehensive consensus agreement and 10% reviewer checks to make sure both researchers are using the same criteria)? Were the characteristics (e.g. age and gender of participants) of the included studies provided in aggregated form? Was the scientific quality of the included studies assessed and documented? Was the scientific quality of the included studies assessed and documented? Was the scientific quality of the included studies used appropriately in formulating conclusions? Are the stated conclusions supported by the data presented?

Analysis of subgroups or subsets
We are yet to determine which subgroup analyses we will conduct.

Contact details for further information
Noa Solomon
noa.solomon@kcl.ac.uk

Organisational affiliation of the review
King's College London
https://www.kcl.ac.uk

Review team members and their organisational affiliations
Miss Noa Solomon. King's College London
Dr Marie-Louise Sharp. King's College London
Professor Nicola Fear.

Collaborators
Dr Helen King. The Open University
Dr Gini Harrison. The Open University
Professor Graham Pike. The Open University

Type and method of review
Systematic review

Anticipated or actual start date
01 April 2019

Anticipated completion date
01 November 2019

Funding sources/sponsors
The Royal Foundation

Conflicts of interest

Language
English

Country
England

Stage of review
Review Ongoing

Subject index terms status
Subject indexing assigned by CRD

Subject index terms
Humans; Mental Health

Date of registration in PROSPERO
28 May 2019

Date of first submission
09 May 2019

Stage of review at time of this submission

Stage	Started	Completed
Preliminary searches	No	No
Piloting of the study selection process	Yes	No
Formal screening of search results against eligibility criteria	No	No
Data extraction	No	No
Risk of bias (quality) assessment	No	No
Data analysis	No	No

The record owner confirms that the information they have supplied for this submission is accurate and complete and they understand that deliberate provision of inaccurate information or omission of data may be construed as scientific misconduct.
The record owner confirms that they will update the status of the review when it is completed and will add publication details in due course.

Versions

28 May 2019
