# Supplementary material for: The mental health and wellbeing of spouses, partners and children of emergency responders: A systematic review
Source: PLoS One. 2022 Jun 15;17(6):e0269659. doi: 10.1371/journal.pone.0269659 (PMC9200352; doi:10.1371/journal.pone.0269659)
Supplement: S1 File — (DOCX) [file pone.0269659.s004.docx]

# Supporting Information – S1 File. – Definitions and Inclusion/Exclusion Criteria

### Inclusion Criteria

1. Empirical studies (using primary data).
2. Studies published in peer-reviewed journals.
3. Studies using quantitative, qualitative, or mixed methods designs assessing mental health and well-being outcomes.
4. Studies published in English.
5. Studies published since 2000.
6. Studies that assessed the families of ER populations including police, firefighters, paramedics, Coastguard, Mountain Rescue, Internet Child Abuse Investigator (ICAT) officers, patrol officers, call-handlers, or other public safety roles (see next section for excluded populations).
7. Studies that measured mental health, including common mental health disorders (depression, and anxiety disorders), post-traumatic stress disorder (PTSD), alcohol problems (hazardous drinking, misuse, abuse, dependence etc.) or substance misuse, stress (traumatic stress reaction, burnout, secondary traumatic stress, compassion fatigue).
8. Studies that measured wellbeing: these were defined to encompass both hedonic and eudemonic traditions including concepts of emotional wellbeing and personal development. Hence encompassing studies that assess factors including: positive relations with others, autonomy, purpose in life, personal growth (Ryff, 1989), social support and relationships (Larson, 1993), satisfaction with life, long-term good emotions and an absence of unpleasant emotions (Diener, 2000) and resilience.
9. Studies that measured work-related measures for wellbeing such as employment status, short/long term sick leave, voluntary resignation and financial outcomes such as debt.

### Exclusion Criteria

1. Papers examining mental health, wellbeing outcomes in families of general emergency department roles (such as nurses or doctors) with no pre-hospital emergency responsibilities.
2. Papers assessing only the physical health of ER families.
