# Supplementary material for: The mental health and wellbeing of spouses, partners and children of emergency responders: A systematic review
Source: PLoS One. 2022 Jun 15;17(6):e0269659. doi: 10.1371/journal.pone.0269659 (PMC9200352; doi:10.1371/journal.pone.0269659)
Supplement: S2 File — (DOCX) [file pone.0269659.s005.docx]

# Supporting Information S2 File. Search Terms

Key search terms related to ‘mental health’, ‘wellbeing’, ‘emergency responders’ and ‘family’ were combined with appropriate Boolean operators.

1. “mental health" OR "psychological distress" OR "mental illness*" OR "mental disorder*" OR "common mental health disorder*" OR anxiety OR "stress disorder*" OR "acute stress" OR traum* OR "post?traumatic stress disorder*" OR “PTSD” or “depress*” OR “alcohol*” OR "substance misuse" OR “diction” OR “burnout” OR “fatigue” or "secondary traumatic stress" or "substance abuse"
2. “well?being OR relationship* OR "social support" OR sleep OR obesity OR appetite OR welfare OR employ* OR volunt* OR resignation OR "long?term sick leave" OR "short?term sick leave" OR finance* OR debt OR socialis*
3. "Emergency Responder*" or police* or "fire?arms officer*" or "community support officer*" or "fire service*" or fire?fighter* or ambulance* or "air ambulance*" or paramedic* or "emergency service*" or "search and rescue" or "mountain rescue" or coast?guard or "call handler*" or RNLI or pre?hospital or "emergency responder*" or "emergency medical technician*" or "rescue worker*" or "public safety officer*" or "emergency service responder*" or "blue?light service*" or “99”
4. “Famil*” OR “wives” OR “wife OR spouse*” OR “intimate partner*” OR “co-habitating partner*” OR “couple*” OR “child*” OR “adolescen*” OR “youth*” OR “dependent*” OR “parent*”.
