# Supplementary material for: The mental health and wellbeing of spouses, partners and children of emergency responders: A systematic review
Source: PLoS One. 2022 Jun 15;17(6):e0269659. doi: 10.1371/journal.pone.0269659 (PMC9200352; doi:10.1371/journal.pone.0269659)
Supplement: S3 File — (DOCX) [file pone.0269659.s006.docx]

# Supporting Information S3 File. Quality Analysis Method

A quality appraisal tool was adapted to critically evaluate the articles by drawing on relevant questions regarding recruitment, method and design, from the Consolidated Criteria for Reporting Qualitative Research (COREQ) (Tong, Sainsbury & Craig, 2007) and ‘The Quality Assessment Tool for Observational Cohort and Cross-Sectional Studies’ (NIH Quality Assessment Tools).

The following guideline questions were used for qualitative studies:

1. Is there a clear rationale and theoretical basis for the study?
2. Were all the subjects selected or recruited from the same or a similar population (including the same time-period)?
3. Were inclusion and / or exclusion criteria for being in the study clearly stated, pre-specified and applied uniformly to all participants?
4. Were there enough participants in the qualitative stud, i.e. was data saturation reached?
5. Were there at least two coders coding the data?
6. Did the study report clearly defined major and minor themes supported by quotes from the interviews?

The following guideline questions were used for quantitative studies:

1. Was the research question or objective in this paper clearly stated?
2. Was another sampling method used besides convenience sampling?
3. Were all the subjects selected or recruited from the same or a similar population (including the same time-period)?
4. Were inclusion and / or exclusion criteria for being in the study clearly stated, pre-specified and applied uniformly to all participants?
5. Was a sample size justification, power description, or variance and effect estimates provided?
6. Were the outcome measures clearly defined, valid, reliable, and implemented consistently across all study participants?

Each question carried ‘1’ if criteria was met and ‘0’ if not, scores were then added up and studies were classified as either being of:

(a) poor quality: total scores of 0, 1, or 2;

(b) fair quality: total scores of 3, or 4;

(c) good quality: total scores of 5, or 6.

One researcher (NS) scored the studies. Issues of quality are noted in the supporting information (S1 Table.) and commented upon in the discussion.
